# Signaling via a CD28/CD40 chimeric costimulatory antigen receptor (CoStAR™), targeting folate receptor alpha, enhances T cell activity and augments tumor reactivity of tumor infiltrating lymphocytes

**DOI:** 10.3389/fimmu.2023.1256491

**Published:** 2023-11-07

**Authors:** Milena Kalaitsidou, Owen R. Moon, Martina Sykorova, Leyuan Bao, Yun Qu, Sujita Sukumaran, Michael Valentine, Xingliang Zhou, Veethika Pandey, Kay Foos, Sergey Medvedev, Daniel J. Powell Jr, Akshata Udyavar, Eric Gschweng, Ruben Rodriguez, Mark E. Dudley, Robert E. Hawkins, Gray Kueberuwa, John S. Bridgeman

**Affiliations:** ^1^ Department of Research, Instil Bio, Dallas, TX, United States; ^2^ Ovarian Cancer Research Center, Division of Gynecologic Oncology, Department of Obstetrics and Gynecology, Perelman School of Medicine, University of Pennsylvania, Philadelphia, PA, United States

**Keywords:** tumor infiltrating lymphocytes (TIL), folate receptor (FR), scFv, chimeric receptor, CD28, CD40, costimulation

## Abstract

Transfer of autologous tumor infiltrating lymphocytes (TIL) to patients with refractory melanoma has shown clinical efficacy in a number of trials. However, extending the clinical benefit to patients with other cancers poses a challenge. Inefficient costimulation in the tumor microenvironment can lead to T cell anergy and exhaustion resulting in poor anti-tumor activity. Here, we describe a chimeric costimulatory antigen receptor (CoStAR) comprised of FRα-specific scFv linked to CD28 and CD40 intracellular signaling domains. CoStAR signaling alone does not activate T cells, while the combination of TCR and CoStAR signaling enhances T cell activity resulting in less differentiated T cells, and augmentation of T cell effector functions, including cytokine secretion and cytotoxicity. CoStAR activity resulted in superior T cell proliferation, even in the absence of exogenous IL-2. Using an *in vivo* transplantable tumor model, CoStAR was shown to improve T cell survival after transfer, enhanced control of tumor growth, and improved host survival. CoStAR could be reliably engineered into TIL from multiple tumor indications and augmented TIL activity against autologous tumor targets both *in vitro* and *in vivo*. CoStAR thus represents a general approach to improving TIL therapy with synthetic costimulation.

## Introduction

Tumors are frequently infiltrated with immune cells which mount an ongoing response to the neoplastic tissue. The interplay between immune cell populations and tumor cells determines the outcome of the disease, and in refractory metastatic disease the immune response is insufficient to adequately control the spread of the tumor. Recent advances in development of checkpoint inhibition immunotherapy have shown that shifting the balance of inhibitory and stimulatory signals within a tumor can result in tumor control (1).

Tumor infiltrating lymphocytes (TILs) are critical to the ongoing surveillance and immune response to neoplasia; a proportion of these TILs consist of polyclonal T cells with antitumor reactivity (2). The ability to extract TILs from a surgically resected tumor, expand them *ex vivo*, and infuse them back into patients has shown clinical efficacy in cases of metastatic cutaneous melanoma (3–11). TIL therapy has been clinically explored in other indications with some evidence of efficacy reported, but with generally overall lower response rates observed (12–19).

The antitumor reactivity of TILs is mediated by the interaction of the T cell receptor (TCR) and peptide major histocompatibility complex (pMHC) on the surface of the tumor cell. Full and sustained T cell activation requires a primary signal (signal 1) driven by TCR recognition of pMHC. Signal 1 is enhanced by secondary costimulatory signals (signal 2) generally provided by antigen-presenting cells (APCs). Costimulation through signal 2 drives enhanced cytokine production, clonal expansion, and upregulation of anti-apoptotic proteins in TILs (20). Despite their ability to recognize and eliminate tumor cells, without robust costimulation, TILs in the tumor microenvironment can be sub-optimally activated and may undergo adaptive tolerance or apoptosis (21). Indeed, multiple studies demonstrate that TIL contain a proportion of cells in an exhausted state, that may in fact be characteristic of neoantigen reactive T cells (22–24).

Insufficient costimulation of TIL can occur through several routes: senescent T cells infiltrating tumors can lose CD28 expression (25), tumors do not express high levels of costimulatory ligands (26), the tumor microenvironment lacks APCs that effectively activate antitumor immunity (27, 28), and multiple cell types in the tumor microenvironment express inhibitory ligands that suppress TIL activity and antagonize costimulatory signals (29). Further studies have elucidated that the presence of antigen-presenting cell niches in the TME are important for the function of immune checkpoint blockade, further highlighting the importance of optimal TIL costimulation (30).

Evidence for the power of engineered costimulation in the cell therapy field comes from the successful application of 2^nd^ generation chimeric antigen receptors (CARs) containing costimulatory domains, which have enhanced *in vivo* persistence compared to 1^st^ generation counterparts (31). Such improvements have led to 2^nd^ generation CARs being approved as a therapeutic agent in a number of hematological indications. Extending this principle to TIL, data from preclinical models suggests that TIL antitumor activity may also be augmented with additional costimulatory signals (32–35).

Herein, we describe a costimulatory antigen receptor (CoStAR) consisting of a folate receptor alpha (FRα) specific scFv, fused to the signaling domains from CD28 and CD40. FRα represents a suitable target for such a strategy, as it is overexpressed in several cancer indications including ovarian, lung and renal cancer (36–39). CoStAR delivers synthetic costimulation upon engagement with FRα, only in the presence of TCR signaling. CoStAR significantly augmented effector function of T cells responding to T cell agonists and pMHC, enhanced tumor control in a solid cancer xenograft model, even in the absence of exogenous IL-2 support, and enhanced activity of TIL derived from multiple indications.

## Materials and methods

Commercial antibodies and reagents used for flow cytometry are described in the **Supplemental Table**. Antibodies were anti-human unless specifically described otherwise. For detection of anti-FRα CoStAR molecule, recombinant FRα-Fc antigen or 19.1 anti-MOV19 anti-idiotype antibody was used. 19.1 anti-MOV19 was kindly provided by Instituto Nazionale dei Tumori (INT)-Milan (40).

### Immunohistochemistry

Immunohistochemistry was performed by Mosaic Laboratories. The FRα antibody (mouse clone BN3.2) was purchased from Leica Biosystems and used at 1.5 µg/mL. The mouse (IgG)1ĸ clone MOPC-31C antibody was purchased from BD Pharmingen (San Diego, CA) and used at 1.5 µg/mL. Staining was evaluated by a pathologist. The FRα (mouse clone BN3.2) assay was evaluated on a semi-quantitative scale, and the percentage of cancer cells or normal cells (specificity section only) staining at each of the following four levels was recorded: 0 (no staining), 1+ (weak staining), 2+ (moderate staining) and 3+ (strong staining). A tumor or normal sample was considered positive if at least 1% of cells demonstrated positive expression. An H-Score was calculated based on the summation of the product of percent of cells stained at each staining intensity using the following equation: (3 x % cells staining at 3+) + (2 x % cells staining at 2+) + (1 x % cells staining at 1+).

### Media and solution formulation

T cell medium (TCM) was formulated using Roswell Park Memorial Institute (RPMI) 1640 medium supplemented with GlutaMAX™ and 25mM 4-(2-hydroxyethyl)-1-piperazineethanesulfonic acid (HEPES), 10% fetal bovine serum (FBS), 1% penicillin/streptomycin, and 50 mM 2-mercaptoethanol. Complete RPMI medium (cRPMI) was formulated using RPMI 1640 supplemented with GlutaMAX™ and 25mM HEPES, 10% FBS and 1% penicillin/streptomycin. PEF solution was formulated using Dulbecco’s phosphate-buffered saline (DPBS), 2mM ethylenediaminetetraacetic acid (EDTA), and 0.5% FBS. PBS-EDM consists of PBS, 0.3% Collagenase, 300 U/mL DNase and 2 mM CaCl_2_.

### Cell lines and constructs

174 x CEM.T2 (from herein termed T2), NCI-H508 (from herein termed H508), NIH : OvCAR-3 (from herein termed OvCAR-3) and K562 were all obtained from ATCC. BA/F3 were obtained from DSMZ. In some experiments parental cell lines were lentivirally engineered to express a membrane bound OKT3 molecule consisting of the OSM protein signal peptide, OKT3 heavy chain variable region (BAA11539.1 amino acids 20-138), a x3 (G4S) linker, OKT3 light chain variable region (CAA01594.1, amino acids 23-129) and HLA-A transmembrane region (AAA59637.1, amino acids 169-206).

FRα CoStAR consists of the variable heavy (VH) and variable light (VL) chains of the anti-folate receptor antibody MOV19 ( (41) and CAA68252.1 and CAA68253.1). The heavy and light chains were joined with a S(G4S)x3 linker region in the orientation of VH-VL, with addition of an OSM signal peptide. The signaling domain consists of a fusion of CD28 (NP_006130.1: residues 21-220) fused directly to CD40 (NP_001241.1: residues 216-277), linked to the scFv via an AAA(GSG)x2 motif. All nucleotide sequences were codon optimized for human expression.

The sequences of the CEA TCR and MART-1 TCR have been previously described (42, 43). TCRs were cloned with a murine β constant domain to permit detection versus endogenous TCR expression. All constructs were cloned into pSF.Lenti plasmid from Oxford Genetics (OG269) with either an EF1α or MND promoter. All gene synthesis was undertaken by Azenta or Genscript.

### Generation of modified T cells

T cells were isolated from peripheral-blood mononuclear cells (PBMCs) using EasySep ™ Human T cell isolation kit (StemCell Technologies), activated with Dynabeads™ Human T-Activator CD3/CD28 (Thermo Fisher) or TransAact (Miltenyi) according to manufacturers’ instructions, with 200 IU/mL of recombinant human interleukin-2 (IL-2). 24-48 hours later, T cells were transduced at an MOI of 5 or 10 with lentiviruses encoding anti-MART1 or CEA TCR to generate TCR-Td, anti-FRα CoStAR molecule for CoStAR-Td, both TCR and anti-FRα CoStAR molecule for TCR.CoStAR-Td, or left non-transduced for the Non-Td group.

In experiments where cells were sorted for transgene expression, cells were harvested between days 5 and 9 after transduction and stained for TCR expression with anti-mouse TCR β BV421 (Biolegend) and/or CoStAR molecule expression with recombinant human FOLR1-Fc fusion protein (Acro Biosystems) and anti-human IgG (γ chain) PE secondary antibody (Sigma). Transgene expressing cells were enriched using the MACSQuant Tyto and expanded in G-Rex plates following a rapid expansion protocol (REP).

### Flow cytometry

Cells were stained with viability dye and Fc receptor blocking reagent according to manufacturer’s instructions. Staining for CoStAR was performed by addition of rhFOLR1-Fc (Acro Biosystems) or anti-Idiotype antibody, followed by anti-human IgG (γ chain) PE secondary (Sigma-Aldrich), or anti-mouse IgG1-PE for detection of FRα or anti-Idiotype respectively, followed by addition of any cell surface antigen specific antibodies.

Tail vain bleeds on days 14 and 21 underwent RBC lysis using diluted 10x RBC lysis buffer (Biolegend) according to the manufacturer’s protocol. Samples were incubated with murine Fc receptor block and viability stain, followed by anti-CD3, anti-CD4, anti-CD8, and anti-murine TCRβ. CoStAR was detected as described above. Following staining, cells were washed and resuspended in PEF for analysis. Samples were acquired on either a BD LSR Fortessa X-20 or NovoCyte 3005 Flow Cytometer System, and analyzed using FlowJo_v10 or Novoexpress software, respectively.

### Rapid expansion protocol

T cells were cultured in TCM with 200 IU/mL IL-2, 30 ng/mL anti-CD3 (Miltenyi MACS GMP CD3 pure) and irradiated allogeneic PBMCs at a ratio of 1: 200 T cell to PBMC, for 14 days with regular media changes. Cells were cryopreserved in CryoStor solution (Sigma-Aldrich).

### Peptide pulsing

Both parental T2 and T2.FRα were incubated with serial 10-fold dilutions of the indicated peptides cRPMI for 1 hour prior to coculture. Non-Td, TCR-Td, CoStAR-Td, and TCR.CoStAR-Td T cells were added to prepared plates with peptide pulsed T2 cells at an effector to target ratio of 1:1. Coculture plates were incubated at 37°C for 20 hours following which supernatants were collected for cytokine analysis.

### Quantification of cytokine secretion

Supernatants harvested from cocultures were assessed using IFNγ ELISA (Biolegend) or MesoScale Discovery (MSD) Custom Human Biomarkers Kit or V-Plex Proinflammatory Panel 1 Human Kits according to the manufacturer’s instructions. The plates were quantified using a MESO QuickPlex SQ 120 reader and analyzed on the MSD WorkBench software (version 4.0.13).

### Cytotoxicity assays

Non-Td or CoStAR-Td T cells were cocultured with the indicated BA/F3 target cell lines at the indicated E:T ratio for five days before staining of the samples with murine anti-CD45 BV785 antibodies (Biolegend) and enumeration of residual cells target cells made using CountBright™ absolute counting beads (Thermo Fisher) and acquisition on a LSR Fortessa X-20 cytometer (BD Biosciences).

### *In vivo* studies

#### Staging, dosing, sample collection and monitoring of mice

1x10^7^ H508.Luc.GFP.FRα cells were subcutaneously injected on the left flank of 6-week-old female NSG mice. When average tumor volume reached between 0.2 - 0.3 cm^3^, mice were randomized into treatment groups and the following day (day 0) given tail vein IV injections of PBS (no treatment) or Non-Td, TCR-Td, CoStAR-Td, or TCR.CoStAR-Td T cells. In IL-2-designated groups, mice received subcutaneous IL-2 doses in the right flank on days 0 to 7. Tumor growth was assessed by digital caliper measurements, and mice were weighed at the same time. When tumor was undetectable, a caliper measurement of 0.001 cm^3^ was recorded. Mice were sacrificed at tumor volume limits (10% tumor volume by mouse body weight) or if clinical condition reached an accordance with the animal science procedures act (ASPA) 1986 and the project license. Live tail vein bleeds were collected on days 14 and 21 in ethylenediaminetetraacetic acid (EDTA)-containing capillary tubes.

For PDX study HGSOC tumor tissue was orthotopically implanted into NSG mice and engraftment followed with 2D ultrasound monitoring. Rolling enrolment into the study was based on consistent tumor growth over three weeks, at which point mice were evenly distributed into five different groups. At the time of enrolment, mice were injected intraperitoneally with FRα CoStAR-Td or Non-Td TIL at a dose of 1x10^7^ TIL. Mice in all groups were injected IP with 5 μg of IL-2 on the day of enrolment, and then again every other day for one week post TIL-injection. Blood was collected five days after the third IL-2 injection via a submandibular bleed. TIL and IL-2 injections were repeated every two weeks for a total of three TIL injections per mouse. Mice were monitored for tumor burden via ultrasound on a weekly basis, and euthanized based on declining body condition score criteria (approved by IACUC). Survival times were recorded.

#### Statistical analysis

All statistical analyses were performed using GraphPad PRISM 9.0.2. For comparison of multiple groups, a mixed-effects analysis was performed using a two-way ANOVA with Sidak’s multiple comparisons test. For coculture assays with solFRα a two-way ANOVA with Tukey’s multiple comparisons test was used. For *in vivo* studies mixed-effects analysis was performed using a 1-way or 2-way ANOVA with Tukey’s test for multiple comparisons. To evaluate survival, a log-rank test with Bonferroni correction for multiple testing was performed. For EC50 values nonlinear regression curves were fitted with a log (agonist) versus response (3 parameters). Comparisons of best fit LogEC50 values were calculated and analyzed by Friedman statistical test with Dunn’s multiple comparisons. Cytokines were compared using 2-way ANOVA.

#### TIL production

On day 1 digested tumor was seeded at 0.5-1x10^6^ viable cells in 2 mL TCM containing 10 μg/mL gentamycin, 0.25 μg/mL amphotericin B, 50 μg/mL vancomycin and 3000 IU/mL rhIL-2. On days 3 and 4 TILs were transduced with lentiviral particles to an MOI of 5 in a total volume of 2.5 mL. In some experiments, TILs were activated on day 1 with TransAct (Miltenyi) according to manufacturer’s instructions. On day 8 the total volume was doubled by addition of fresh TCM containing 20 μg/mL gentamicin 0.5 μg/mL amphotericin B 100 μg/mL vancomycin and 6000 IU/mL IL-2. REPs were established on day 10 in G-Rex (6M) plates.

Cryopreserved Non-Td and CoStAR-Td TILs were thawed and rested overnight in 300 IU/mL IL-2 prior to assay set up or thawed and rested for 3 days in 3000 IU/mL IL-2, washed and rested without IL-2 overnight prior to assay set up. Autologous tumor digests were thawed, diluted 1 x 10^6^/mL and plated into 96-well U-bottom plates. In some experiments CD45 depletion was performed to enrich for tumor using a human CD45+ depletion kit (StemCell Technologies) according to manufactures instructions. CoStAR-Td or Non-Td TILs were added to plates to an effector TIL:digest cell ratio of 1:1. Cocultures with BA/F3 derived engineered target cell lines were set up an E:T of 8:1. Plates were cultured at 37°C in a 5% CO2 humidified incubator for 16-24 hours, 100 μL volume of supernatants were collected, aliquoted and cytokine secretion performed using Mesoscale Discovery (MSD) immunoassay, or IFNγ ELISA (Biolegend).

## Results

**Figure 1:**
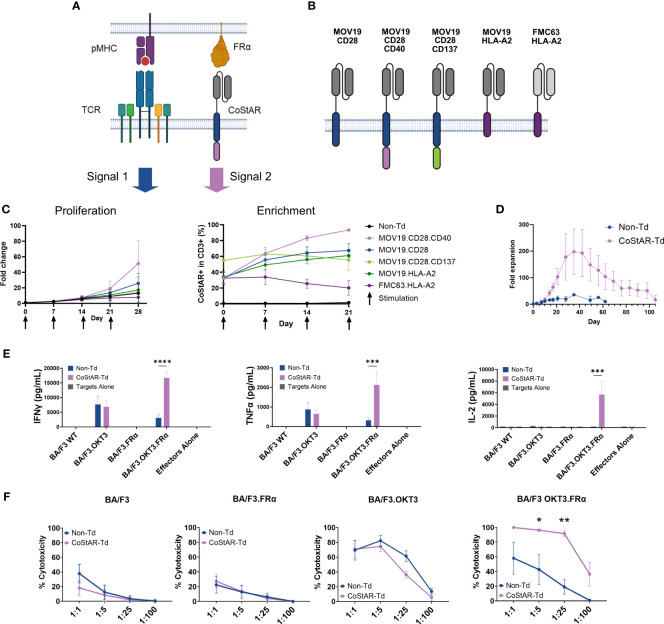
Synthetic costimulation through CoStAR enhances T cell cytolytic activity and cytokine secretion. **(A)** Schematic representation of TCR and CoStAR engagement with pMHC and FRα respectively. **(B)** Schematic representation of CoStAR constructs containing combinations of MOV19 or FMC63 ScFvs, CD28 extracellular, transmembrane and cytoplasmic region, CD40 or CD137 cytoplasmic domains or the non-signaling HLA-A2 transmembrane domain. **(C)** T cells from four healthy donors were transduced with the indicated CoStARs and incubated with OvCAR-3.OKT3 at an effector: target (E:T) of 8:1 and proliferation of T cells and CoStAR+ enrichment monitored over 4 successive restimulations with additional tumor cells (averages of four donors performed in triplicate with SD shown). **(D)** CoStAR-transduced and non-transduced T cells were cocultured with BA/F3.OKT3.FRα cells in the presence of 200 IU/mL IL-2 at Day 0, and T cell counts made at 3 and/or 7-day intervals for 105 days (averages of three donors performed in duplicate with SEM shown). CoStAR transduced and non-transduced or cells were cocultured with WT BA/F3 or BA/F3 cells expressing OKT3, FRα or OKT3 + FRα for 16 hours before analysis of coculture supernatant for IFNγ, IL-2 and TNF **(E)** and cytotoxicity **(F)** of target cells measured at varying E:T ratios at day 5 by counting residual BA/F3 cells using a murine anti-CD45 antibody (averages of three donors performed in duplicate with SEM shown). *P < 0.05, **P < 0.01, ***P <0.001, ****P < 0.0001 by two-way ANOVA with Sidak’s multiple comparisons. Figure created with BioRender.com.

T cell receptor (TCR) signals (Signal 1) mediated through peptide major histocompatibility complex (pMHC) can synergize with costimulatory signal (Signal 2) to enhance T cell survival, proliferation and effector function. We hypothesized that costimulation could be mediated through synthetic costimulation via a chimeric costimulatory receptor consisting of an antigen binding region fused to the signaling domain from a costimulatory receptor (**Figure 1A**). To test this hypothesis, we generated a panel of CoStARs with varying scFv and costimulatory domains (**Figure 1B**).

The anti-FRα CoStARs consist of a MOV19-derived scFv fused via a glycine-serine linker to the extracellular and transmembrane domains of CD28 (amino acids 21-179), followed by the cytoplasmic domain(s) of CD28 (amino acids 180-220), CD28 and CD40 (amino acids 216-277) or CD28 and CD137 (amino acids 209-255). Additional control non-signaling CoStARs were generated by fusing MOV19 or the αCD19 scFv FMC63 to the HLA-A2 transmembrane domain (amino acids 274-313) (**Figure 1B**).

T cells from four healthy donors were transduced with the respective CoStAR construct lentiviruses and normalized for transduction level ranging between 32 to 55% across all constructs. T cells were challenged weekly at an E:T ratio of 8:1 with the FRα^+^ cell line OvCAR-3, engineered to express OKT3 (OvCAR-3.OKT3) in the presence of 200 IU/mL IL-2. Proliferation and CoStAR enrichment was monitored at 7-day intervals, at which time point the T cells were restimulated with OvCAR-3.OKT3 cells (**Figure 1C**). Non-transduced cells expanded by an average of 13.2-fold over the 4 restimulations, while the control, non-FRα targeting FMC63.HLA-A2 CoStAR expanded by 7.9-fold. Of the FRα targeted CoStARs, the lowest fold expansion was observed with the CD28.CD137 fusion (14.3-fold) and the MOV19.HLA-A2 construct (17.3-fold). MOV19.CD28 expanded by 25.9-fold, however, the most striking observation was that elicited by the CD28.CD40 fusion CoStAR which expanded by an average of 51.3-fold (p = 0.0049 vs FMC63.HLA-A2) (left panel, **Figure 1C**; **Supplementary Figure 1A**). Enrichment of MOV19 CoStAR transduced cells was observed, with the CD28.CD40 fusion CoStAR displaying the most robust and efficient enrichment and being the only receptor approaching near pure enrichment (average of 93% CoStAR^+^) by day 21 (right panel, **Figure 1C**). This enrichment of MOV19.CD28.CD40 over 21 days was significantly better than for all other receptors tested except MOV19.CD28 (P > 0.05) (**Supplementary Figure 1B**).

To further explore this optimal CD28.CD40 CoStAR response to FRα upon co-engagement of the TCR we utilized the murine cell line BA/F3 engineered to express OKT3 and FRα. T cells isolated from three healthy donors were transduced to an average transduction rate of 78%. Transduced and non-transduced cells were cocultured with BA/F3.OKT3.FRα at an E:T of 8:1 and maintained by media replenishment with 200 IU/mL IL-2, and fold expansion measured by taking viable cell counts every 2-3 days (**Figure 1D**). Non-Td cells expanded to an average of 36-fold by day 35 before undergoing population contraction, falling below detectable levels by day 62. In contrast, CoStAR transduced cells underwent enhanced proliferation, reaching an average of 198-fold expansion by day 35, and surviving until the end of the assay on day 105.

To test CoStAR modified T cell response to each input signal (i.e., OKT3 and/or FRa), non-transduced and CoStAR transduced T cells were cocultured with individual BA/F3 target lines at an E:T of 1:1. Production of IL-2, TNF, and IFNγ from cocultures was then measured (**Figure 1E**). Production of all three cytokines from transduced and non-transduced T cells alone, or in cocultures with WT BA/F3 or BA/F3.FRα was below the level of detection. Although all three cytokines could be detected in cocultures with BA/F3.OKT3, there was no significant difference between non-transduced and CoStAR transduced cells. However, in cocultures with target cells providing both signal 1 and signal 2 (BA/F3.OKT3.FRα) there was significantly increased production of all three cytokines in CoStAR-Td compared with Non-Td cells.

CoStAR modulation of T cell mediated cytotoxicity was assessed by coculture with engineered target lines at varying E:T ratios (1:1, 1:5, 1:25 and 1:100) and absolute counts of target cells enumerated after 5 days (**Figure 1F**). Killing efficiency between Non-Td and CoStAR-Td was equivalent against WT BA/F3, and more importantly against BA/F3.FRα, the latter indicating that cytotoxicity is not driven by CoStAR engagement alone. Efficient killing of BA/F3.OKT3 cells was observed, with maximal cytotoxicity seen at E:T of 1:5 by CoStAR-Td and Non-Td cells, respectively. Importantly there was no significant difference in killing between CoStAR-Td and Non-Td cells at any ratio tested. In cocultures with BA/F3.OKT3.FRα similar ratio dependent killing responses were seen with transduced and non-transduced cells, however there was significantly enhanced killing of target cells by CoStAR-Td cells at 1:5 and 1:25.

**Figure 2:**
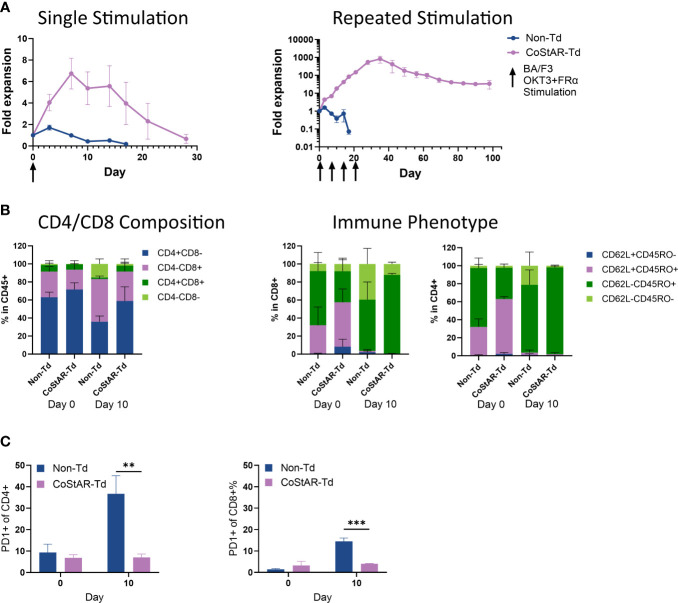
CoStAR enhances T cell proliferation in the absence of exogenous IL-2. **(A)** Control non-transduced (Non-Td) and CoStAR-transduced (CoStAR-Td) T cells were cocultured with BA/F3.OKT3.FRα cells with either a single addition or multiple additions at the indicated time points in the absence of exogenous IL-2, and cell counts made at 3 and/or 7-day intervals for 28 or 105 days respectively. **(B)** Control Non-Td and CoStAR-Td cells were taken at day 0 and day 10 and the CD4 and CD8 composition assessed and the cells immunophenotyped using anti-CD62L and anti-CD45RO antibodies. **(C)** PD1 expression was assessed at day 0 and day 10 in CD4+ and CD8+ T cells. **P < 0.01, ***P <0.001 < 0.001 by two-way ANOVA.

TIL therapy is currently limited by the requirement for high dose IL-2 post-infusion which is associated with significant side effects and patient morbidity. To determine whether CoStAR could mitigate this IL-2 dependence we performed an *in vitro* model of serial stimulation to mimic repeated tumor engagement. To this end, we performed stimulation with single or repeat additions of BA/F3.OKT3.FRα cells at days 0, 7, 14 and 21 in the absence of exogenous IL-2; T cell expansion was assessed by enumeration of total viable cells (**Figure 2A**). Following a single stimulation, Non-Td cell expansion peaked at 1.7-fold by day 3 before contracting to undetectable levels by day 17, whereas CoStAR-Td cell expansion peaked to 6.7-fold by day 7 and survived to day 28. With repeated stimulation, Non-Td cells reached an average fold expansion of 1.5 at day 3 before cell numbers contracted and were undetectable by day 17. In contrast, CoStAR engineered cells were capable of proliferation upon each repeat stimulation with target cells, reaching an average expansion of 829-fold by day 35 and persisting until the end of the assay on day 105.

Both transduced and non-transduced cells started with a CD4^+^ skewed population on day 0 with a skewing towards a more CD8^+^ population by day 10 (non-transduced from an average of 28.3 to 47.3% and CoStAR transduced from 22 to 32.8% CD8^+^) (**Figure 2B**). With respect to differentiation status we observed that the more differentiated CD62L^-^/CD45RO^-^ population had increased from an average of 8.2 to 39.5% and 2.5 to 21.2% in CD8^+^ and CD4^+^ non-transduced T cells respectively; whereas in CoStAR-Td cells this most differentiated subset had only increased from 8.1 to 12.1 in CD8^+^ cells, and decreased from 2.4 to 1.9% in CD4^+^ T cells.

PD1 expression in CD4^+^ cells were, on average, below 10% in both CoStAR-Td and Non-Td cells at day 0. By day 10, following two rounds of stimulation, PD1 expression in CoStAR-Td cells was unchanged, whereas Non-Td cells had significantly elevated levels at an average of 37% (**Figure 2C**). Similar results were observed in CD8^+^ T cells, with significantly elevated levels of PD1 in non-transduced vs transduced cells at day 10. These data demonstrate that CoStAR enhanced proliferation of T cells in an exogenous IL-2 independent manner and, according to PD-1 expression at least, kept the resulting cells in a less exhausted state.

**Figure 3:**
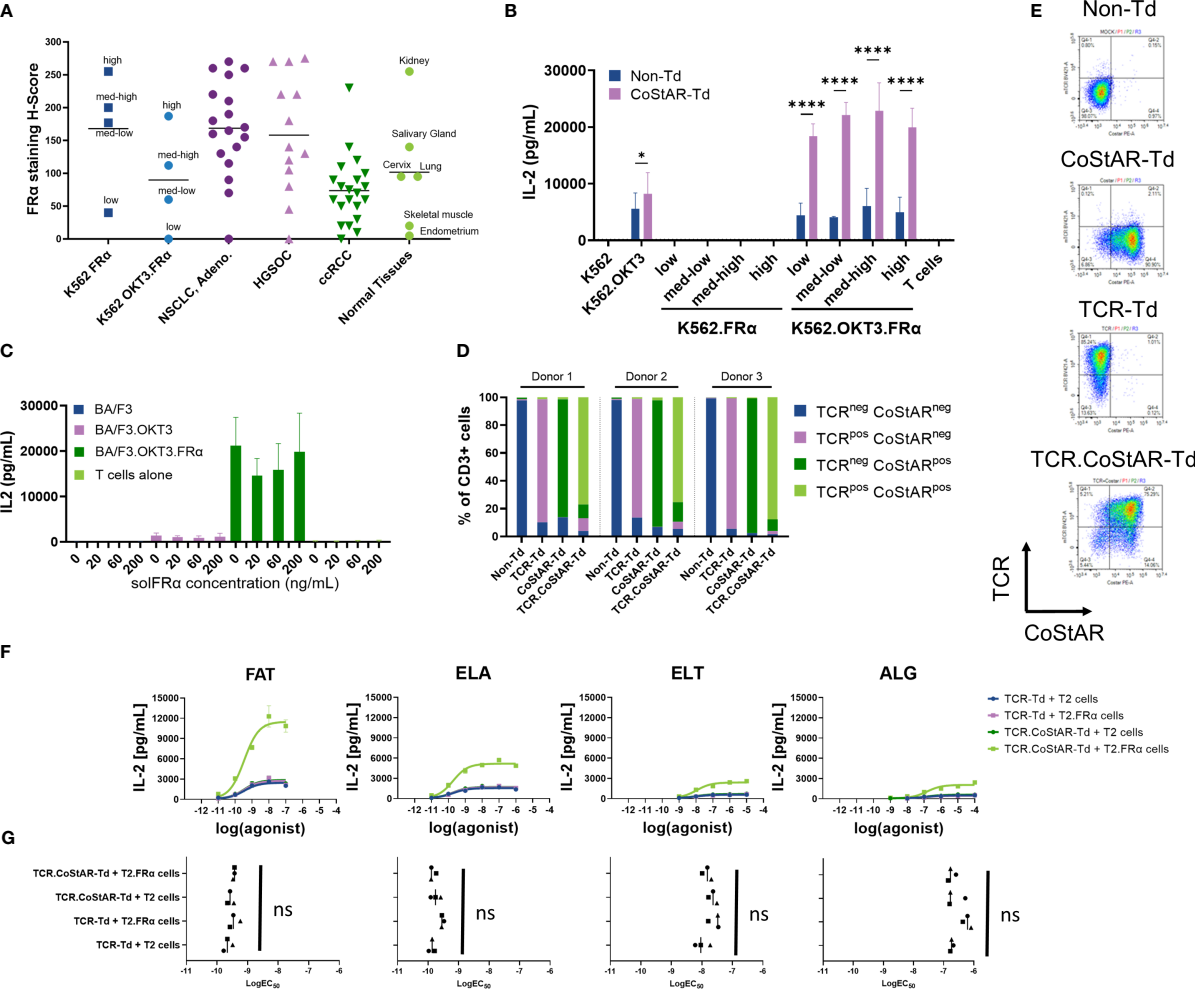
CoStAR engineered cells respond to only membrane bound, but not soluble, FOLR1; and in a dose dependent manner upon signal 1 engagement. **(A)** IHC H-scores from various normal and malignant tissues and engineered K562 cell lines. **(B)** Non-transduced (Non-Td) and CoStAR-transduced (CoStAR-Td) T cells were cocultured at different effector: target (E:T) ratios with K562 parental, K562.OKT3, K562.FRα (low, med-low, med-high, or high), K562.OKT3.FRα (low, med-low, med-high, or high) cells. IL-2 secretion was measured after a 24-hour coculture. Nonsignificant values not shown. Data from 3 donors with 2 technical replicates for each. * P <0.05; **** P <0.0001 by two-way ANOVA with Sidaks multiple comparison. **(C)** CoStAR engineered T cells were cocultured with parental BA/F3, BA/F3.OKT3, BA/F3.FRα or BA/F3.OKT3.FRα in the presence of varying concentrations of soluble FRα (solFRα) protein. Supernatant was collected after a 20-hour coculture and IL-2 measured by MSD. **(D)** Flow cytometric analysis of TCR and anti-FRα CoStAR molecule surface expression in transduced CD3+ cell populations in three donors. **(E)** Representative flow cytometry plots showing the TCR and anti-FRα CoStAR molecule surface expression in transduced CD3+ cell populations. **(F)** TCR-Td T cells (signal 1 only) or TCR.CoStAR-Td T cells (signals 1 and 2) were cocultured with parental T2 or T2.FRα, supernatants were collected after 20 hours of coculture and IL-2 secretion evaluated by MSD assay. One representative donor of three shown with two technical replicates. **(G)** LogEC50 best-fit values were generated by fitting a dose-response curve to cytokine secretion data after T cell coculture with peptide-loaded T2 cell lines. Differences in LogEC50 values between TCR-Td T cells cocultured with T2 parental or T2.FRα both loaded with peptides (signal 1 only) and TCR.CoStAR-Td T cells cocultured with peptide-loaded wild type T2 (signal 1 only) compared with TCR.CoStAR-Td T cells cocultured with peptide-loaded T2.FRα (signals 1 and 2) were evaluated. LogEC50 best-fit values were calculated from secretion of IL-2. Averages from 2 technical replicates in 3 donors were analyzed by Friedman statistical test with Dunn’s multiple comparisons in Graphpad Prism 9.3.0, non-significant differences not shown.

Although FRα is overexpressed in tumor, expression may vary from patient to patient, and across regions within the tumor itself; furthermore, it is known that there is restricted expression on some normal tissue (44, 45). To explore how different levels of FRα in the presence and absence of TCR stimulation affect CoStAR activity, we developed an *in vitro* model system which could be used to further interrogate both off target toxicity and on-target efficacy. K562 cells were engineered to express varying levels of FRα, with or without membrane anchored OKT3. The engineered cell lines, as well as tissue sections from both neoplastic (non-small cell lung cancer adenocarcinoma, high grade serous ovarian cancer and clear cell renal cell carcinoma) and normal tissue (kidney, salivary gland, lung, cervix, skeletal muscle and endometrium), were immunohistologically examined with an IVD approved FRα antibody, and the resulting H-scores calculated (**Figure 3A**). H-score takes into account the proportion and intensity of staining and as such represents a more valid means of assessing expression. The widest range of FRα was observed in HGSOC (H-score range 0 - 275, mean 158). NSCLC had a similar range of FRα expression (range = 0-270; mean = 169), with ccRCC having a more restricted range (range = 0-230; mean = 74) of approximately half that of NSCLC and HGSOC. In normal tissue, the highest observed expression was in normal kidney with a H score of 255, salivary gland, lung and cervix had intermediate H scores (140, 95 and 95 respectively), with skeletal muscle (H=20) and endometrium (H=5) having low H scores. K562.FRα (high) had a similar H score to normal kidney, with K562.FRα (low) having similar expression to skeletal muscle. K562.OKT3.FRα (high) had similar FRα expression to an average NSCLC sample, with the K562.OKT3.FRα (med-high) and (med-low) being similar in FRα expression to an average ccRCC sample. These modified K562 lines were thus indicative of physiological samples for the purposes of the experiment.

To examine the effect of signal 2 alone on CoStAR bearing cells, transduced and non-transduced cells were incubated with K562.FRα target cells expressing different levels of FRα, and cytokine secretion measured after 24 hours (**Figure 3B**, **Supplementary Figures 2A, B**). IL-2 was below the lower limit of detection for all conditions, regardless of FRα expression level or presence of CoStAR. Similar observations were made via analysis of IFNγ (**Supplementary Figure 2A**) and TNF (**Supplementary Figure 2B**). These data highlight the absolute requirement of signal 1 provision for CoStAR mediated enhancement of T cell effector functions.

CoStAR T cell activation by cells expressing varying levels of FRα, with OKT3 as an activating signal was assessed in cocultures with K562.OKT3.FRα for 24 hours (**Figure 3B**, **Supplementary Figures 2A, B**). CoStAR-Td cells demonstrated significantly enhanced IL-2 production (P < 0.0001) in response to K562.OKT3 expressing a range of FRα levels compared to Non-Td cells. Importantly, there was no obvious trend with regards to FRα level and IL-2 production, suggesting that CoStAR enhances responses to even low levels of target antigen. Similar observations were made for IFNγ (**Supplementary Figure 2A**) and TNF (**Supplementary Figure 2B**).

FRα can be shed from the cell surface and is present at elevated levels in cancer patient serum (46). We therefore examined whether soluble (sol)FRα could either costimulate T cells in the presence of a TCR agonist or, conversely, block costimulation mediated through membrane anchored FRα. To this end, we performed cocultures of transduced T cells with BA/F3.OKT3 and BA/F3.OKT3.FRα in the presence of solFRα concentrations reported in ovarian cancer patient serum, as well as at supraphysiological levels (**Figure 3C**). In cocultures with BA/F3.OKT3, no increase in secreted IL-2 was observed in the presence of increasing concentrations of solFRα. To assess the potential impact of solFRα on blocking of CoStAR mediated costimulation through membrane bound FRα, CoStAR-Td T cells were cocultured with BA/F3.OKT3.FRα targets in the presence of increasing concentrations of solFRα. Consistent with previous observations, BA/F3.OKT3.FRα elicited a higher amount of secreted IL-2 from CoStAR T cells compared to BA/F3.OKT3. Importantly, we did not detect inhibition of CoStAR-mediated IL-2 secretion in the presence of BA/F3.OKT3.FRα with increasing concentrations of solFRα. Similar results were obtained by analysis of target cell killing, with solFRα neither potentiating cytotoxicity towards BA/F3 or BA/F3.OKT3, nor blocking cytotoxicity towards BA/F3.OKT3.FRα target cells (**Supplementary Figure 2C**). These data demonstrate that solFRα cannot costimulate CoStAR T cells even at supraphysiological concentration and also does not block CoStAR activity at the physiological concentrations observed in cancer patients.

T cell activation can be mediated through a broad range of agonists, varying both in binding quality and concentration. In most situations these agonist interactions are likely to be less potent than the OKT3 signal used to demonstrate CoStAR activity thus far. We therefore developed a TCR/CoStAR co-transfer model to better understand the relationship between TCR agonism and CoStAR activity. We chose an HLA-A*02-restricted MelanA/MART-1 TCR model for which multiple agonist peptides have been characterized (43, 47–49). T cells from three donors were engineered with either CoStAR or TCR alone, or in combination, and sorted to achieve bulk populations enriched for expression. In all three donors, over 85% expressed anti-MART1 TCR in TCR-Td condition, over 84% expressed anti-FRα CoStAR molecule in the CoStAR-Td condition, and over 75% expressed both anti-MART-1 TCR and anti-FRα CoStAR molecule in the TCR.CoStAR-Td condition gated from CD3^+^ cells (**Figures 3D, E**). T cells were then cocultured with WT or FRα-transduced T2 target cells pulsed with varying concentrations of four different agonist peptides (FATGIGIITV [FAT], ELAGIGILTV [ELA], ELTGIGILTV [ELT] and ALGIGILTV [ALG]) of decreasing agonist activity and cytokine secretion was measured after a 20-hour coculture. These peptide concentrations used spanned the 1x10^-9^/1x10^-10^ M concentrations which have previously been shown to cover the range typical for a naturally presented tumor associated antigen (50).

IL-2 release by activated T cells (TCR-Td or TCR.CoStAR-Td conditions) showed a dose-dependent correlation with the antigen concentration. Also, the intensity of response correlated with described pMHC affinity towards anti-MART1-TCR (43, 47, 48, 51), with FAT peptide generating the strongest response, closely followed by ELA peptide, ELT generating weaker response, and ALG generating the weakest response from the 4 tested peptides (**Figure 3F**, **Supplementary Figures 2E, F**). Consistent with previous observations, CoStAR enhanced IL-2 secretion only in the presence of both signal 1 agonists and FRα. These levels were higher compared to the dose-response curves from cocultures with either TCR-Td cells responding to peptide pulsed T2 or T2.FRα lines or TCR.CoStAR-Td cells responding to peptide pulsed T2 cells. Cocultures without a signal 1 element, between T2.WT or T2.FRα cells and CoStAR Td cells did not induce IL-2 secretion above detectable limits (data not shown). We used dose response curves generated to calculate EC_50_ values for each condition (**Figure 3G**, **Supplementary Figure 2D**). Interestingly, we found that although CoStAR enhanced overall effector function elicited by a number of altered peptide ligands, there was no observable difference in the concentration of peptide required to elicit 50% maximal activation. Thus, CoStAR does not affect the EC_50_ of pMHC engagement mediated through the TCR.

**Figure 4:**
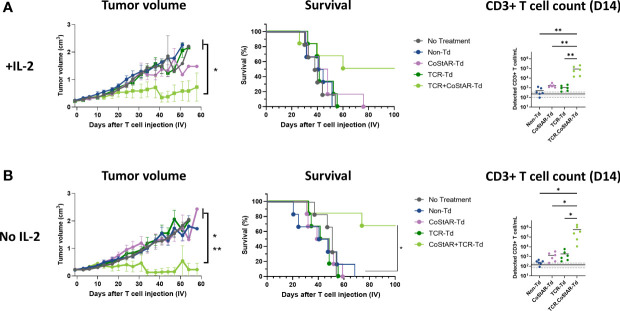
CoStAR enhances tumor control and survival in a murine xenograft model even in the absence of exogenous IL-2 infusions. Tumor volume, survival and peripheral T cell counts were measured in animals receiving **(A)** or not receiving **(B)** exogenous IL-2 administration. Tumor growth was assessed by digital calliper measurements. Survival of mice with established subcutaneous H508.Luc.GFP.FRα tumors was monitored after adoptive cell transfer of Non-Td and CoStAR-Td T cells, and mice were sacrificed at experimental endpoints. Flow cytometric assessment of CD3+ T cells/mL in mouse tail-vein bleeds in all T cell groups with or without supportive IL-2 on day 14. Mean and individual data points shown. Detection of T cells in mice that received no treatment is used to define the detection limit of the assay and is shown as lines on the y axis, solid line = mean and dotted line = SD. T cell counts were compared using one-way ANOVA with Tukey's multiple comparison * P <0.05, **P<0.01. To compare tumor volume mixed-effects model with Tukey’s multiple comparisons test was performed * P <0.05 (No treatment & Non-Td Vs TCR.CoStAR-Td) ** P <0.01 (TCR-Td & CoStAR-Td vs TCR.CoStAR-Td). To evaluate survival, a log-rank test with Bonferroni multiple comparison was used, * P <0.05 (Non-Td & CoStAR Td vs 658 TCR.CoStAR-Td).

To investigate the efficacy of CoStAR-Td T cells *in vivo*, FRα engineered NCI-H508 (H508.Luc.GFP.FRα) cell line, which presents a CEA derived peptide (CEA:691-699 IMIGVLVGV) via HLA-A*02 to a high affinity CEA-specific TCR (S112T TCR (42): was engrafted subcutaneously in NSG mice. PBS or Non-Td, TCR-Td, CoStAR-Td or TCR.CoStAR-Td cells at a 5x10^6^ dose (**Supplementary Figure 3A, B**) were injected into tumor bearing mice on day 0. 5x10^6^ T cells was shown to be the minimum number of cells capable of eliciting tumor control with this donor (P = 0.004 & 0.0075 vs PBS for 1x10^7^ and 5x10^6^, respectively). Administration of TCR.CoStAR-Td T cells from two independent donors alongside exogenous IL-2 led to significantly better control of tumor growth relative to Non-Td in donor 1 (**Figure 4A**, **Supplementary Figure 3C**) and Non-Td, CoStAR-Td, and TCR-Td treatment groups in donor 2 (**Supplementary Figure 3C**). For both donors, CoStAR expression alone did not limit tumor growth without TCR co-expression. At terminal end-points or day 58, the tumor volume of mice treated with TCR.CoStAR-Td T cells was significantly reduced in comparison to control groups across two independent donors (**Supplementary Figure 3D**). Conversely, Non-Td, TCR-Td, and CoStAR-Td treatment groups had no significant differences in tumor size from the PBS group.

Given the mechanism of action of the anti-FRα CoStAR molecule, which promotes *in vitro* T cell expansion in an IL-2 independent manner, we utilized our *in vivo* model to examine whether the anti-FRα CoStAR molecule circumvented the requirement of exogenous IL-2 administration. Tumor engrafted NSG mice were treated with either PBS or Non-Td, TCR-Td, CoStAR-Td or TCR.CoStAR-Td cells at the 5x10^6^ dose in the absence of IL-2 support. Under these conditions, TCR.CoStAR-Td T cells exhibited significantly improved control of tumor growth compared to all other treatment groups from days 0 - 58 with 4/6 mice having undetectable tumors on day 58 (**Figure 4B**, **Supplementary Figure 3D**). 3/6 and 4/6 mice treated with TCR.CoStAR-Td T cells survived until the end of the study at day 99 with and without supportive IL-2 administration, respectively (**Figure 4B**). In comparison, when mice received supportive IL-2, and Non-Td, CoStAR-Td, or TCR-Td T cells, mice reached their tumor volume limits by days 52, 76, and 55, respectively (**Figure 4A**). When exogenous IL-2 was withheld, Non-Td, CoStAR-Td, and TCR-Td treatment groups reached experimental endpoints by days 69, 59, and 55, respectively (**Figure 4B**) demonstrating CoStAR mediated co-stimulation improves T cell efficacy *in vivo* irrespective of supportive IL-2, and does not circumvent TCR specificity.

In line with the improved *in vitro* T cell expansion endowed to transduced T cells by CoStAR (**Figure 2**), TCR.CoStAR-Td T cells showed increased levels of T cells when compared with all other treatment groups on day 14. With supportive IL-2 administration the concentration of CD3 T cells/mL was 8.68 (± SD 7.86) x 10^4^ in the TCR.CoStAR-Td group which was significantly higher than for Non-Td, CoStAR-Td and TCR-Td groups whose concentrations were 0.52 (± SD 0.46) x 10^3^, 1.73 (± SD 0.78) x 10^3^, and 1.07 (± SD 0.64) x10^3^ CD3 T cells/mL, respectively (**Figure 4A**). Similar effects were seen with a second donor in the presence of exogenous IL-2 (**Supplementary Figure 3E**). In the absence of IL-2 support, the detected concentrations of CD3 T cells in the TCR.CoStAR-Td treatment group was again significantly elevated with 2,237.7-, 447.8-, and 319.7-fold increases of CD3 T cells/mL of murine peripheral blood relative to Non-Td, CoStAR-Td, and TCR-Td treatment groups, respectively (**Figure 4B**). When taken together, these data demonstrate that anti-FRα CoStAR molecule increased peripheral blood T cell levels, efficacy, and survival only in the presence TCR-pMHC-mediated signal 1 and does so irrespectively of exogenous IL-2 support.

**Figure 5:**
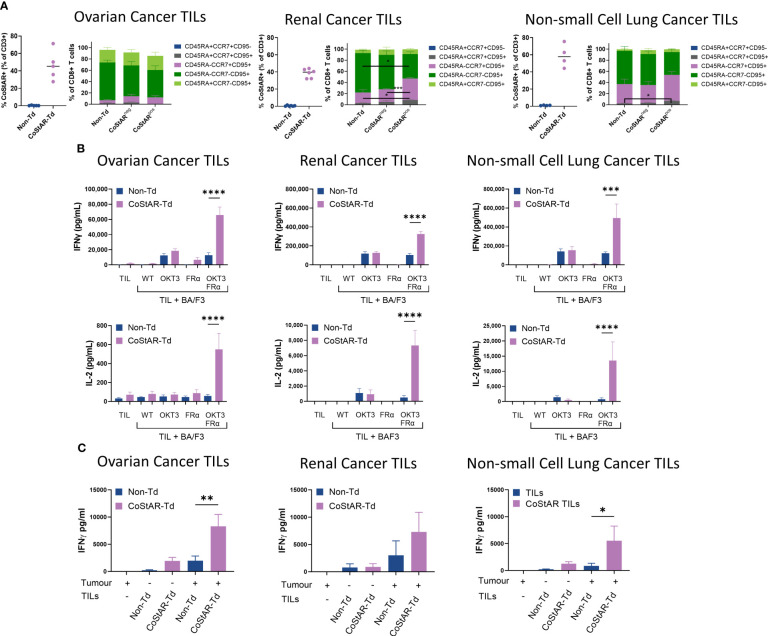
CoStAR can be efficiently expressed in TIL and enhances effector function in response to autologous tumor. **(A)** TIL from ovarian, renal and non-small cell lung cancer tumors were successfully transduced with CoStAR and differentiation status assessed by flow cytometry (Tn (CD45RA+CCR7+CD95-), Tscm (CD45RA+CCR7+CD95+), Tcm (CD45RA-CCR7+CD95+), Tem (CD45RA-CCR7-CD95+), and Tte (CD45RA+CCR7-CD95+)). * P <0.05, *** P <0.001 by two-way ANOVA with Tukey’s multiple comparison. **(B)** Control non-transduced (Non-Td) or CoStAR transduced (CoStAR-Td) TIL were cocultured with WT BA/F3 or BA/F3 cells expressing OKT3, FRα or OKT3 + FRα before measurement of IL-2 and IFNγ. **(C)** Control Non-Td and CoStAR-Td TIL were cocultured with autologous tumor digest before measurement of IFNγ. * P <0.05, ** P <0.01, *** P<0.001, **** P <0.0001 by one way ANOVA with Sidaks multiple comparisons test.

To verify the functional attributes and benefits of CoStAR in clinically relevant TIL, five ovarian, six renal, and four lung tumor samples were transduced with CoStAR to average efficiencies of 46, 38, and 59%, respectively (**Figure 5A**). The phenotype of TIL within the non-transduced populations as well as the CoStAR^neg^ and CoStAR^pos^ cells within the transduced populations were assessed to determine whether CoStAR expression affected the phenotype of TIL (**Figure 5A**). To this end, cells were stained with antibodies to define the following populations: Tn (CD45RA^+^CCR7^+^CD95-), Tscm (CD45RA^+^CCR7^+^CD95^+^), Tcm (CD45RA-CCR7^+^CD95^+^), Tem (CD45RA-CCR7-CD95^+^), and Tte (CD45RA^+^CCR7-CD95^+^). Ovarian TIL had a dominant Tem phenotype, followed by Tte, and a smaller proportion of Tcm, with no significant differences observed between the three populations analyzed. Renal TIL tended towards a less Tte, and a more Tcm skewed phenotype than ovarian TIL, with CoStAR^pos^ cells harboring a significantly higher frequency of Tcm than Non-Td TIL (P=0.0417) or corresponding CoStAR^neg^ TIL (P=0.0009). TIL derived from lung tumors on average had a propensity towards a more Tcm phenotype than either the renal or ovarian TIL, but retaining a more Tem phenotype overall. The only significant difference observed was within the Tscm population where a difference existed between Non-Td and CoStAR^pos^ cells (P=0.0331), although this population represented a small proportion overall. Even though differences were seen within some individual populations within indications, overall TIL phenotypes between Non-Td, CoStAR^neg^ and CoStAR^pos^ populations looked remarkably similar.

To assess the biological activity and benefit of CoStAR in TIL, cytokines were analyzed from overnight cocultures of CoStAR-Td and Non-Td TIL from the three indications with WT parental BA/F3 cells or BA/F3 expressing OKT3, FRα or OKT3+FRα (**Figure 5B**). Irrespective of CoStAR expression, TIL from the three indications produced low or undetectable levels of IFNγ and IL-2 either alone or in response to WT BA/F3 and BA/F3.FRα. As expected, both IFNγ and IL-2 was detectable from cocultures with BA/F3.OKT3 and did not significantly differ between Td and Non-Td TIL. This suggests that CoStAR does not negatively affect the TIL’s natural ability to respond to TCR signaling. Importantly, CoStAR-Td produced higher levels of IFNγ (ovarian: 5.1-fold (P=0.0219); renal: 3.1-fold (P=0.0016); lung 4.0-fold) and IL-2 (ovarian: 9.3-fold; renal: 15.0-fold; lung: 18.3-fold) than Non-Td TIL in response to BA/F3.OKT3.FRα. Similar trends were seen with IL-8, IL-13 and TNF but not IL-4 (**Supplementary Figure 4A–C**). We also analyzed IL-1b, IL-6, IL-10 and IL-12 which were not above the level of detection or at biologically relevant concentrations (data not shown).

Reactivity of Non-Td and CoStAR-Td TIL towards autologous tumor was assessed to ascertain the potential enhancement of anti-tumor activity by the CoStAR molecule against ovarian, renal and lung tumor expressing FRα (**Figure 5C**). FRα could be readily detectable in ovarian, renal and non-small cell lung cancer digests (**Supplementary Figure 4D**). CoStAR-Td and Non-Td TIL were cocultured with autologous digest overnight at an E:T ratio of 1:1 for cytokine secretion analysis. IFNγ secretion from digest alone was undetectable with minimal amounts of background IFNγ observed from CoStAR-Td and Non-Td TIL alone. In the presence of autologous digest, CoStAR-Td TIL from all three indications produced more IFNγ compared to Non-Td TIL (ovarian: 4.3-fold; renal: 2.4-fold and lung: 6.4-fold). These data demonstrate that CoStAR can boost anti-tumor responses of TIL across multiple FRα expressing tumors.

**Figure 6:**
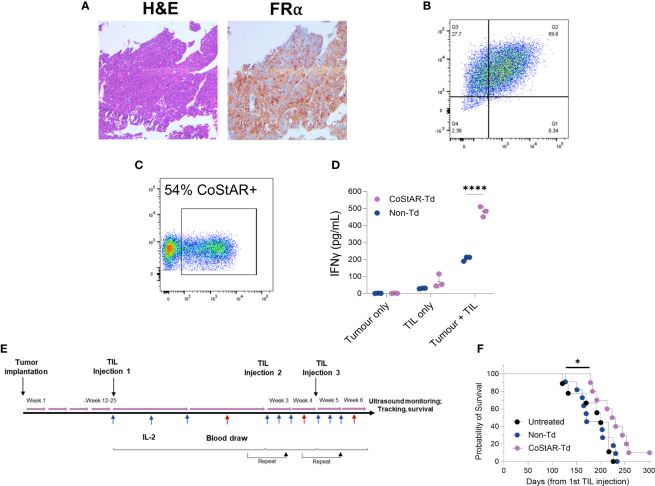
CoStAR improves survival in a PDX model of ovarian cancer. Orthotopically implanted ovarian tumor isolated from engrafted mice were stained and analysed by IHC for H&E and FRα **(A)**, and digested and analysed by flow cytometry for EpCAM and FRα **(B)**. TIL isolated from the same tumor specimen were transduced with CoStAR **(C)**, before mixing with autologous tumor and IFNγ analysed by ELISA **(D)**. **** P <0.0001 by two-way ANOVA with Sidaks multiple comparisons test. Schematic representation of the tumor implantation and TIL administration regimen for the PDX model shown **(E)**. Survival curve shows the comparison of the probability of survival of mice treated with TILs at N=10 to 11 mice per group **(F)**. * P<0.05 by Log Rank test.

We next sought to investigate the anti-tumor activity of CoStAR engineered TIL in an *in vivo* setting. To this end tumor tissue from a patient with high grade serous ovarian cancer (HGSOC) was orthotopically implanted into NSG mice to generate a patient derived xenograft (PDX) model. IHC and flow cytometric analysis of engrafted tumor showed broad expression of FRα (**Figures 6A, B**). TIL from the same patient were isolated and engineered with CoStAR to 54% transduction efficiency (**Figure 6C**). These TIL elicited effector activity towards autologous tumor, which was significantly enhanced (P<0.0001) in the presence of CoStAR (**Figure 6D**). Upon tumor engraftment mice were intraperitoneally injected with a first dose of 1x10^7^ Non-Td or CoStAR-Td TIL, followed by additional 2 injections at 2- and 4- weeks post 1^st^ infusion of 1x10^7^ TIL, with IL-2 provided during the first week after TIL injection (**Figure 6E**). Even in this extremely challenging *in vivo* model of TIL activity we observed a significantly delayed time to death in animals given CoStAR-Td cells compared to those given Non-Td cells (P=0.0174), with 1 animal surviving to >300 days post 1^st^ TIL injection (**Figure 6F**).

## Conclusion

Numerous trials have demonstrated the clinical feasibility and striking response rates achieved via the autologous transfer of TIL to patients who have failed all other treatment options. Although response rates have been consistently impressive in metastatic cutaneous melanoma there is scope to improve lower response rates seen in trials of TIL in other indications. The CoStAR platform described herein offers an opportunity to enhance the therapeutic efficacy of TIL by augmenting the natural signals generated through endogenous TCR-pMHC interactions and leveraging signals mediated through a chimeric costimulatory signaling domain consisting of CD28 and CD40.

Although there are sporadic reports of CD40 being expressed in certain T cell subsets (52, 53), it is not typically considered to be a classical T cell costimulatory receptor. However, several of the tumor necrosis superfamily receptors are expressed in T cells (e.g. CD27, CD134 and CD137) that utilize the same signaling pathways to induce activation (54). Furthermore, both constitutively active CD40 (55), and a chimeric Myd88-CD40 receptor have been shown to be a potent drivers of T cell expansion (56). Indeed, there is also evidence that incorporation of CD40 into a CAR configuration is at least comparable to existing 41BB containing CARs (57).

We made four particularly striking observations which outlines the potential therapeutic advantages of the CoStAR platform. First, CoStAR is dependent on TCR signals to mediate activation, with no activity (cytotoxicity or cytokine production) observed in response to CoStAR binding alone, even at FRα expression levels equivalent to those at the highest physiological levels. This is in concordance with the typical behavior of natural costimulation. FRα has a distribution of tissue expression which, although deemed tumor specific, is also expressed at low levels on some normal tissue. Preclinical and clinical studies with FRα targeted monoclonal antibodies (58) and FRα specific CARs (59) have demonstrated good safety profiles. The data herein gives us confidence that any on-target/off-tumor binding events will not drive pathology.

Second, CoStAR does not respond to soluble FRα. We show that even at supraphysiological concentrations of soluble FRα there is no enhancement in activity of CoStAR engineered T cells responding to signal 1 alone. This could be explained by a mechanism in which CoStAR triggering requires the antigen to be presented in an immobilized state.

Third, soluble FRα does not block the activity of CoStAR towards membrane anchored FRα, even at supraphysiological concentrations. Although quite surprising, it is in concordance with the behavior of chimeric antigen receptors, whose activity is not negatively impacted by high concentrations of soluble antigen (60). Studies suggest that soluble free proteins have more rapid binding kinetics compared to those immobilized and may explain in part this observation (61).

The ability of CoStAR to support T cell expansion in the absence of exogenous IL-2 is an intriguing and unexpected observation with translatable benefits for TIL therapy where post-infusion IL-2 remains a significant cause of treatment morbidity and barrier to the wider exploitation of this therapeutic intervention. The increased production of autocrine IL-2 production by T cells coactivated by CoStAR may be one explanation for this observation. Another possibility is that costimulation separately through CD28 (62) or CD40 (63, 64) induces anti-apoptotic signaling pathways which may play a role in the survival of CoStAR engineered cells in the absence of IL-2. Considering the very high systemic concentrations of IL-2 normally given to TIL patients, we do not expect the concentrations produced by CoStAR-TIL to be a potential cause of patient morbidity, nonetheless monitoring this will be an important component of a phase I study of CoStAR-TIL.

Herein we also demonstrate that CoStAR does not alter the activation threshold of T cells via modulation of the EC50. Viola and Lanzavecchia in their seminal paper (65) demonstrated that CD28 costimulation lowered the TCR activation threshold. However, this is less clear with other costimulatory receptors. Dushek and colleagues have demonstrated that CD28, as well as various TNF receptor superfamily members, can have modest effects of lowering EC50 of T cell activation which can be contextually dependent, but that CD2 which acts as an adhesion/costimulatory receptor can have a much larger effect on EC50 (66, 67). Thus, the effect of costimulation can be impacted by a combination of avidity and signaling. As chimeric receptors can uncouple this effect, by altering the biophysical parameters associated with engagement of the receptor, it may be unwise to directly compare the effects of CoStAR with historical analysis of native costimulatory receptors. In support of this, CARs incorporating costimulatory domains do not lower the activation threshold compared to first generation CARs (60).

The modular nature of the CoStAR platform allows for substantial flexibility, with the targeting of a diverse array of target antigens for broad applicability across an array of tumor indications, and will be explored in more granularity in future studies. Herein we further show that the applicability of this technology allows for enhanced reactivity of TIL towards autologous FRα expressing tumor types (lung, renal and ovarian) tumor both *in vitro* and *in vivo* and as such sets the scene for FRα CoStAR being evaluated clinically in a phase 1 trial.

## Data availability statement

The datasets presented in this article are not readily available because the data is proprietary to Instil Bio. Requests to access the datasets should be directed to josh.johnson@instilbio.com.

## Ethics statement

The studies involving humans were approved by local ethics review board and relevant REC approval for UK consented participants. The studies were conducted in accordance with the local legislation and institutional requirements. The participants provided their written informed consent to participate in this study. The animal study was approved by Animal Science Procedures Act (ASPA) 1986 and Establishment and Project license guidelines. The project license was reviewed and approved by the local animal and welfare review body (AWERB). The study was conducted in accordance with the local legislation and institutional requirements.

## Author contributions

MK: Conceptualization, Formal Analysis, Methodology, Supervision, Writing – review & editing, Investigation, Validation. OM: Formal Analysis, Investigation, Methodology, Validation, Writing – review & editing. MS: Formal Analysis, Investigation, Methodology, Writing – review & editing. LB: Methodology, Investigation, Writing – review & editing. YQ: Formal Analysis, Investigation, Methodology, Writing – review & editing. SS: Formal Analysis, Investigation, Methodology, Writing – review & editing. MV: Investigation, Writing – review & editing. XZ: Investigation, Writing – review & editing. VP: Investigation, Writing – review & editing, Formal Analysis, Methodology. KF: Formal Analysis, Investigation, Methodology, Writing – review & editing. SM: Methodology, Investigation, Writing – review & editing. DP: Writing – review & editing, Resources, Supervision. AU: Supervision, Writing – review & editing, Data curation, Formal Analysis, Project administration. EG: Data curation, Formal Analysis, Supervision, Writing – review & editing, Methodology. RR: Formal Analysis, Methodology, Supervision, Writing – review & editing, Project administration. MD: Supervision, Writing – review & editing. RH: Supervision, Writing – review & editing, Conceptualization, Funding acquisition, Methodology, Project administration. GK: Conceptualization, Methodology, Project administration, Supervision, Writing – review & editing, Data curation, Formal Analysis. JB: Conceptualization, Funding acquisition, Methodology, Project administration, Supervision, Validation, Visualization, Writing – original draft, Writing – review & editing.

## Supplementary material

Supplementary Figure 1 A CD28.CD40-based CoStAR offers optimal antigen mediated costimulation of T cells. **(A)** T cells from four healthy donors were transduced with the indicated CoStARs and incubated with OvCAR-3.OKT3 at an E:T of 8:1 and proliferation of T cells **(A)** and CoStAR+ enrichment **(B)** monitored over four successive restimulations with additional tumor cells. Area under the curve (AUC) for expansion was calculated for each construct and significance calculated using a Friedman test: ** P <0.01. Fold enrichment of cells expressing each CoStAR was determined between days 0 and 21 and significance calculated using a one-way ANOVA with Tukey’s multiple comparison test: * P <0.05, ** P <0.01, *** P <0.001, **** P<0.0001. **(C)** BA/F3 cells were left non-transduced or engineered to express a membrane anchored OKT3 scFv and/or human FRα to provide signals 1 and/or 2 respectively. Flow cytometric analysis performed by staining with anti-FRα-PE or detection of OKT3 fluorescent tag (GFP). Figure created with Biorender.com.

Supplementary Figure 2 CoStAR engineered cells respond to membrane bound, but not soluble, FRα; and in a dose dependent manner upon signal 1 engagement. Non-transduced (Non-Td) and CoStAR transduced (CoStAR-Td) T cells were cocultured at different effector: target (E:T) ratios with K562 parental, K562.OKT3, K562.FRα (low, med-low, med-high, or high), K562.OKT3.FRα (low, med-low, med-high, or high) cells. IFNγ **(A)** and TNF **(B)** secretion was measured after a 24-hour coculture. Averages from 3 donors in duplicate shown. * P <0.05; **** P <0.0001 by two-way ANOVA with Sidaks multiple comparison. **(C)** CoStAR-Td T cells were cocultured with BA/F3, BA/F3.OKT3 or BA/F3.OKT3.FRα cells at a 1:1 E:T in the presence of varying concentrations of soluble FRα (solFRα) protein ratio and residual tumor cells counted after 20 hours via murine anti-CD45 staining. **(D)** LogEC50 best-fit values were generated by fitting a dose-response curve to cytokine secretion data after T cell coculture with peptide-loaded T2 cell lines. Differences in LogEC50 values between TCR-Td T cells cocultured with T2 parental or T2.FRα both loaded with peptides (signal 1 only) and TCR.CoStAR-Td T cells cocultured with peptide-loaded wild type T2 (signal 1 only) compared with TCR.CoStAR-Td T cells cocultured with peptide-loaded T2.FRα (signals 1 and 2) were evaluated. LogEC50 best-fit values were calculated from secretion of IFNγ and TNF Averages from 2 technical replicates in 3 donors were analyzed by Friedman statistical test with Dunn’s multiple comparisons in Graphpad Prism 9.3.0. TCR-Td T cells (signal 1 only) or TCR.CoStAR-Td T cells (signals 1 and 2) were cocultured with parental T2 or T2.FRα, supernatants were collected after 20 hours of coculture and IL-2, IFNγ and TNF secretion evaluated by MSD assay. Two representative donors of three shown **(E, F)** performed in duplicate.

Supplementary Figure 3 CoStAR enhances T cell persistence and tumor control in a murine xenograft model even in the absence of exogenous IL-2 infusions. Mice were subcutaneously implanted with H508 tumors and administered with varying numbers of non-transduced or CEA-TCR engineered T cells at the indicated numbers. **(A)** Tumor volumes were measured by digital caliper measurements up to day 58. **(B)** Survival of animals was also monitored. **(C)** Tumor volumes were measured twice weekly by digital caliper measurements up to day 58. **(D)** Comparison of tumor volumes at day 58 in treatment groups from 2 donors with, and one donor without, administration of supportive IL-2. **(E)** Flow cytometric assessment of CD3+ T cells/mL in mouse tail-vein bleeds in all T cell groups with or without supportive IL-2 on day 14. Mean and individual data points shown. CD3+ levels were compared using one-way ANOVA with Tukey’s multiple comparison. To compare tumor volume mixed-effects model with Tukey’s multiple comparisons test was performed* P <0.05 (No treatment & Non-Td Vs TCR.CoStAR-Td) ** P <0.01 (TCR-Td & CoStAR-Td vs TCR.CoStAR-Td). To compare survival a log-rank test with Bonferroni correction for multiple corrections was used to determine the adjusted significance threshold, * P <0.05 (Non-Td & CoStAR Td vs TCR.CoStAR-Td), ** P <0.01.

Supplementary Figure 4 CoStAR can be efficiently expressed in TIL and enhances effector function in response to autologous tumor. TIL from ovarian **(A)**, renal **(B)** and non-small cell lung **(C)** were successfully transduced with CoStAR and cocultured with WT BA/F3 or BA/F3 cells expressing OKT3, FRα or OKT3 + FRα before measurement of IL-4, IL-8, IL-13 and TNF **(A–C)**. FRα expression was assessed by flow cytometry in the CD2- population of ovarian digested tumor or CD45- population of renal and non-small cell lung cancer digests **(D)**. * P <0.05, ** P <0.01 by one-way ANOVA with Sidak’s multiple comparisons test.

## References

[1] Larkin J Chiarion-Sileni V Gonzalez R Grob J-J Rutkowski P Lao CD. Five-year survival with combined nivolumab and ipilimumab in advanced melanoma. New Engl J Med (2019) 381(16):1535–46. doi: 10.1056/NEJMoa1910836 31562797

[2] Gokuldass A Draghi A Papp K Borch TH Nielsen M Westergaard MCW. Qualitative analysis of tumor-infiltrating lymphocytes across human tumor types reveals a higher proportion of bystander Cd8(+) T cells in non-melanoma cancers compared to melanoma. Cancers (Basel) (2020) 12(11):3344. doi: 10.3390/cancers12113344 33198174 PMC7696049

[3] Besser MJ Shapira-Frommer R Treves AJ Zippel D Itzhaki O Schallmach E. Minimally cultured or selected autologous tumor-infiltrating lymphocytes after a lympho-depleting chemotherapy regimen in metastatic melanoma patients. J Immunotherapy (2009) 32(4):415–23. doi: 10.1097/CJI.0b013e31819c8bda 19342963

[4] Dudley ME Gross CA Langhan MM Garcia MR Sherry RM Yang JC. Cd8+ Enriched “Young” Tumor infiltrating lymphocytes can mediate regression of metastatic melanoma. Clin Cancer Res (2010) 16(24):6122–31. doi: 10.1158/1078-0432.Ccr-10-1297 PMC2978753 20668005

[5] Rosenberg SA Yang JC Sherry RM Kammula US Hughes MS Phan GQ. Durable complete responses in heavily pretreated patients with metastatic melanoma using T-cell transfer immunotherapy. Clin Cancer Res (2011) 17(13):4550–7. doi: 10.1158/1078-0432.Ccr-11-0116 PMC3131487 21498393

[6] Ellebaek E Iversen TZ Junker N Donia M Engell-Noerregaard L Met Ö. Adoptive cell therapy with autologous tumor infiltrating lymphocytes and low-dose interleukin-2 in metastatic melanoma patients. J Transl Med (2012) 10:169. doi: 10.1186/1479-5876-10-169 22909342 PMC3514199

[7] Besser MJ Shapira-Frommer R Itzhaki O Treves AJ Zippel DB Levy D. Adoptive transfer of tumor-infiltrating lymphocytes in patients with metastatic melanoma: intent-to-treat analysis and efficacy after failure to prior immunotherapies. Clin Cancer Res (2013) 19(17):4792–800. doi: 10.1158/1078-0432.Ccr-13-0380 23690483

[8] Nguyen LT Saibil SD Sotov V Le MX Khoja L Ghazarian D. Phase ii clinical trial of adoptive cell therapy for patients with metastatic melanoma with autologous tumor-infiltrating lymphocytes and low-dose interleukin-2. Cancer Immunol Immunother (2019) 68(5):773–85. doi: 10.1007/s00262-019-02307-x PMC11028227 30747243

[9] Pillai M Hawkins R Jiang Y Lorigan P Thistlethwaite F Thomas M. Treatment outcomes with unselected autologous tumor infiltrating lymphocytes (Tils) in patients (Pts) with checkpoint inhibition-refractory advanced cutaneous melanoma. Annals of Oncology (2021) 32(5):S882. doi: 10.1016/j.annonc.2021.08.1443

[10] Chesney J Lewis KD Kluger H Hamid O Whitman E Thomas S. Efficacy and safety of lifileucel, a one-time autologous tumor-infiltrating lymphocyte (Til) cell therapy, in patients with advanced melanoma after progression on immune checkpoint inhibitors and targeted therapies: pooled analysis of consecutive cohorts of the C-144-01 study. J Immunother Cancer (2022) 10(12):e005755. doi: 10.1136/jitc-2022-005755 36600653 PMC9748991

[11] Rohaan MW Borch TH van den Berg JH Met Ö Kessels R Geukes Foppen MH. Tumor-infiltrating lymphocyte therapy or ipilimumab in advanced melanoma. N Engl J Med (2022) 387(23):2113–25. doi: 10.1056/NEJMoa2210233 36477031

[12] Ratto GB Zino P Mirabelli S Minuti P Aquilina R Fantino G. A randomized trial of adoptive immunotherapy with tumor-infiltrating lymphocytes and interleukin-2 versus standard therapy in the postoperative treatment of resected nonsmall cell lung carcinoma. Cancer (1996) 78(2):244–51. doi: 10.1002/(sici)1097-0142(19960715)78:2<244::Aid-cncr9>3.0.Co;2-l 8673999

[13] Stevanović S Draper LM Langhan MM Campbell TE Kwong ML Wunderlich JR. Complete regression of metastatic cervical cancer after treatment with human papillomavirus-targeted tumor-infiltrating T cells. J Clin Oncol (2015) 33(14):1543–50. doi: 10.1200/jco.2014.58.9093 PMC4417725 25823737

[14] Stevanović S Helman SR Wunderlich JR Langhan MM Doran SL Kwong MLM. A phase ii study of tumor-infiltrating lymphocyte therapy for human papillomavirus-associated epithelial cancers. Clin Cancer Res (2019) 25(5):1486–93. doi: 10.1158/1078-0432.Ccr-18-2722 PMC6397671 30518633

[15] Figlin RA Thompson JA Bukowski RM Vogelzang NJ Novick AC Lange P. Multicenter, randomized, phase iii trial of cd8(+) tumor-infiltrating lymphocytes in combination with recombinant interleukin-2 in metastatic renal cell carcinoma. J Clin Oncol (1999) 17(8):2521–9. doi: 10.1200/jco.1999.17.8.2521 10561318

[16] Pedersen M Westergaard MCW Milne K Nielsen M Borch TH Poulsen LG. Adoptive cell therapy with tumor-infiltrating lymphocytes in patients with metastatic ovarian cancer: A pilot study. Oncoimmunology (2018) 7(12):e1502905. doi: 10.1080/2162402x.2018.1502905 30524900 PMC6279323

[17] Kverneland AH Pedersen M Westergaard MCW Nielsen M Borch TH Olsen LR. Adoptive cell therapy in combination with checkpoint inhibitors in ovarian cancer. Oncotarget (2020) 11(22):2092–105. doi: 10.18632/oncotarget.27604 PMC7275789 32547707

[18] Creelan BC Wang C Teer JK Toloza EM Yao J Kim S. Tumor-infiltrating lymphocyte treatment for anti-Pd-1-resistant metastatic lung cancer: A phase 1 trial. Nat Med (2021) 27(8):1410–8. doi: 10.1038/s41591-021-01462-y PMC8509078 34385708

[19] Chandran SS Somerville RPT Yang JC Sherry RM Klebanoff CA Goff SL. Treatment of metastatic uveal melanoma with adoptive transfer of tumour-infiltrating lymphocytes: A single-centre, two-stage, single-arm, phase 2 study. Lancet Oncol (2017) 18(6):792–802. doi: 10.1016/s1470-2045(17)30251-6 28395880 PMC5490083

[20] Liechtenstein T Dufait I Lanna A Breckpot K Escors D. Modulating co-stimulation during antigen presentation to enhance cancer immunotherapy. Immunol Endocr Metab Agents Med Chem (2012) 12(3):224–35. doi: 10.2174/187152212802001875 PMC3428911 22945252

[21] Nurieva R Wang J Sahoo A. T-cell tolerance in cancer. Immunotherapy (2013) 5(5):513–31. doi: 10.2217/imt.13.33 PMC5103631 23638746

[22] Siddiqui I Schaeuble K Chennupati V Fuertes Marraco SA Calderon-Copete S Pais Ferreira D. Intratumoral tcf1(+)Pd-1(+)Cd8(+) T cells with stem-like properties promote tumor control in response to vaccination and checkpoint blockade immunotherapy. Immunity (2019) 50(1):195–211.e10. doi: 10.1016/j.immuni.2018.12.021 30635237

[23] Zheng L Qin S Si W Wang A Xing B Gao R. Pan-cancer single-cell landscape of tumor-infiltrating T cells. Science (2021) 374(6574):abe6474. doi: 10.1126/science.abe6474 34914499

[24] Lowery FJ Krishna S Yossef R Parikh NB Chatani PD Zacharakis N. Molecular signatures of antitumor neoantigen-reactive T cells from metastatic human cancers. Science (2022) 375(6583):877–84. doi: 10.1126/science.abl5447 PMC8996692 35113651

[25] Huff WX Kwon JH Henriquez M Fetcko K Dey M. The evolving role of cd8(+)Cd28(-) immunosenescent T cells in cancer immunology. Int J Mol Sci (2019) 20(11):2810. doi: 10.3390/ijms20112810 31181772 PMC6600236

[26] Capece D Verzella D Fischietti M Zazzeroni F Alesse E. Targeting costimulatory molecules to improve antitumor immunity. J BioMed Biotechnol (2012) 2012:926321. doi: 10.1155/2012/926321 22500111 PMC3303883

[27] Fu C Jiang A. Dendritic cells and cd8 T cell immunity in tumor microenvironment. Front Immunol (2018) 9:3059. doi: 10.3389/fimmu.2018.03059 30619378 PMC6306491

[28] Bandola-Simon J Roche PA. Dysfunction of antigen processing and presentation by dendritic cells in cancer. Mol Immunol (2019) 113:31–7. doi: 10.1016/j.molimm.2018.03.025 PMC6173666 29628265

[29] Ayala-Mar S Donoso-Quezada J González-Valdez J. Clinical implications of exosomal pd-L1 in cancer immunotherapy. J Immunol Res (2021) 2021:8839978. doi: 10.1155/2021/8839978 33628854 PMC7886511

[30] Duraiswamy J Turrini R Minasyan A Barras D Crespo I Grimm AJ. Myeloid antigen-presenting cell niches sustain antitumor T cells and license pd-1 blockade *via* cd28 costimulation. Cancer Cell (2021) 39(12):1623–1642.e20. doi: 10.1016/j.ccell.2021.10.008 PMC8861565 34739845

[31] Savoldo B Ramos CA Liu E Mims MP Keating MJ Carrum G. Cd28 costimulation improves expansion and persistence of chimeric antigen receptor-modified T cells in lymphoma patients. J Clin Invest (2011) 121(5):1822–6. doi: 10.1172/jci46110 PMC3083795 21540550

[32] Townsend SE Allison JP. Tumor rejection after direct costimulation of cd8+ T cells by B7-transfected melanoma cells. Science (1993) 259(5093):368–70. doi: 10.1126/science.7678351 7678351

[33] Hernandez-Chacon JA Li Y Wu RC Bernatchez C Wang Y Weber JS. Costimulation through the cd137/4-1bb pathway protects human melanoma tumor-infiltrating lymphocytes from activation-induced cell death and enhances antitumor effector function. J Immunother (2011) 34(3):236–50. doi: 10.1097/CJI.0b013e318209e7ec PMC3063939 21389874

[34] Leem G Park J Jeon M Kim ES Kim SW Lee YJ. 4-1bb co-stimulation further enhances anti-pd-1-mediated reinvigoration of exhausted Cd39(+) Cd8 T cells from primary and metastatic sites of epithelial ovarian cancers. J Immunother Cancer (2020) 8(2):e001650. doi: 10.1136/jitc-2020-001650 33335029 PMC7745695

[35] Beckermann KE Hongo R Ye X Young K Carbonell K Healey DCC. Cd28 costimulation drives tumor-infiltrating T cell glycolysis to promote inflammation. JCI Insight (2020) 5(16):e138729. doi: 10.1172/jci.insight.138729 32814710 PMC7455120

[36] Assaraf YG Leamon CP Reddy JA. The folate receptor as a rational therapeutic target for personalized cancer treatment. Drug Resistance Updates (2014) 17(4):89–95. doi: 10.1016/j.drup.2014.10.002 25457975

[37] Fisher RE Siegel BA Edell SL Oyesiku NM Morgenstern DE Messmann RA. Exploratory study of 99mtc-ec20 imaging for identifying patients with folate receptor-positive solid tumors. J Nucl Med (2008) 49(6):899–906. doi: 10.2967/jnumed.107.049478 18483093

[38] Notaro S Reimer D Fiegl H Schmid G Wiedemair A Rössler J. Evaluation of folate receptor 1 (Folr1) mrna expression, its specific promoter methylation and global DNA hypomethylation in type I and type ii ovarian cancers. BMC Cancer (2016) 16:589. doi: 10.1186/s12885-016-2637-y 27485273 PMC4971744

[39] Scaranti M Cojocaru E Banerjee S Banerji U. Exploiting the folate receptor a in oncology. Nat Rev Clin Oncol (2020) 17(6):349–59. doi: 10.1038/s41571-020-0339-5 32152484

[40] Pupa SM Bazzini P Menard S Colnaghi MI. Network of idiotypic and anti-idiotypic antibodies related to the ovarian carcinoma-associated folate binding protein. Anticancer Res (1992) 12(5):1565–70. 1332580

[41] Figini M Obici L Mezzanzanica D Griffiths A Colnaghi MI Winter G. Panning phage antibody libraries on cells: isolation of human fab fragments against ovarian carcinoma using guided selection. Cancer Res (1998) 58(5):991–6. 9500461

[42] Parkhurst MR Joo J Riley JP Yu Z Li Y Robbins PF. Characterization of genetically modified T-cell receptors that recognize the cea:691-699 peptide in the context of Hla-A2.1 on human colorectal cancer cells. Clin Cancer Res (2009) 15(1):169–80. doi: 10.1158/1078-0432.Ccr-08-1638 PMC3474199 19118044

[43] Madura F Rizkallah PJ Holland CJ Fuller A Bulek A Godkin AJ. Structural basis for ineffective T-cell responses to mhc anchor residue-improved “Heteroclitic” Peptides. Eur J Immunol (2015) 45(2):584–91. doi: 10.1002/eji.201445114 PMC4357396 25471691

[44] Weitman SD Lark RH Coney LR Fort DW Frasca V Zurawski VR. Distribution of the folate receptor gp38 in normal and Malignant cell lines and tissues. Cancer Res (1992) 52(12):3396–401. 1596899

[45] Weitman SD Weinberg AG Coney LR Zurawski VR Jennings DS Kamen BA. Cellular localization of the folate receptor: potential role in drug toxicity and folate homeostasis. Cancer Res (1992) 52(23):6708–11. 1330299

[46] Kurosaki A Hasegawa K Kato T Abe K Hanaoka T Miyara A. Serum folate receptor alpha as a biomarker for ovarian cancer: implications for diagnosis, prognosis and predicting its local tumor expression. Int J Cancer (2016) 138(8):1994–2002. doi: 10.1002/ijc.29937 26595060

[47] Clement M Ladell K Ekeruche-Makinde J Miles JJ Edwards ES Dolton G. Anti-cd8 antibodies can trigger Cd8+ T cell effector function in the absence of tcr engagement and improve peptide-mhci tetramer staining. J Immunol (2011) 187(2):654–63. doi: 10.4049/jimmunol.1003941 PMC3145095 21677135

[48] Ekeruche-Makinde J Clement M Cole DK Edwards ES Ladell K Miles JJ. T-cell receptor-optimized peptide skewing of the T-cell repertoire can enhance antigen targeting. J Biol Chem (2012) 287(44):37269–81. doi: 10.1074/jbc.M112.386409 PMC3481325 22952231

[49] Valmori D Fonteneau JF Lizana CM Gervois N Liénard D Rimoldi D. Enhanced generation of specific tumor-reactive Ctl in vitro by selected melan-a/mart-1 immunodominant peptide analogues. J Immunol (1998) 160(4):1750–8. doi: 10.4049/jimmunol.160.4.1750 9469433

[50] Bossi G Gerry AB Paston SJ Sutton DH Hassan NJ Jakobsen BK. Examining the presentation of tumor-associated antigens on peptide-pulsed T2 cells. Oncoimmunology (2013) 2(11):e26840. doi: 10.4161/onci.26840 24482751 PMC3894244

[51] Bridgeman JS Sewell AK Miles JJ Price DA Cole DK. Structural and biophysical determinants of Aβ T-cell antigen recognition. Immunology (2012) 135(1):9–18. doi: 10.1111/j.1365-2567.2011.03515.x 22044041 PMC3246648

[52] Bourgeois C Rocha B Tanchot C. A role for cd40 expression on cd8+ T cells in the generation of cd8+ T cell memory. Science (2002) 297(5589):2060–3. doi: 10.1126/science.1072615 12242444

[53] Vaitaitis GM Waid DM Yussman MG Wagner DH Jr. Cd40-mediated signalling influences trafficking, T-cell receptor expression, and T-cell pathogenesis, in the nod model of type 1 diabetes. Immunology (2017) 152(2):243–54. doi: 10.1111/imm.12761 PMC5588813 28542921

[54] Dostert C Grusdat M Letellier E Brenner D. The tnf family of ligands and receptors: communication modules in the immune system and beyond. Physiol Rev (2019) 99(1):115–60. doi: 10.1152/physrev.00045.2017 30354964

[55] Levin N Weinstein-Marom H Pato A Itzhaki O Besser MJ Eisenberg G. Potent activation of human T cells by mrna encoding constitutively active cd40. J Immunol (2018) 201(10):2959–68. doi: 10.4049/jimmunol.1701725 30305327

[56] Collinson-Pautz MR Chang WC Lu A Khalil M Crisostomo JW Lin PY. Constitutively active myd88/cd40 costimulation enhances expansion and efficacy of chimeric antigen receptor T cells targeting hematological Malignancies. Leukemia (2019) 33(9):2195–207. doi: 10.1038/s41375-019-0417-9 PMC6756044 30816327

[57] Levin-Piaeda O Levin N Pozner S Danieli A Weinstein-Marom H Gross G. The intracellular domain of cd40 is a potent costimulatory element in chimeric antigen receptors. J Immunother (2021) 44(6):209–13. doi: 10.1097/cji.0000000000000373 34010245

[58] Shimizu T Fujiwara Y Yonemori K Koyama T Sato J Tamura K. First-in-human phase 1 study of morab-202, an antibody-drug conjugate comprising farletuzumab linked to eribulin mesylate, in patients with folate receptor-A-positive advanced solid tumors. Clin Cancer Res (2021) 27(14):3905–15. doi: 10.1158/1078-0432.Ccr-20-4740 33926914

[59] Song DG Ye Q Carpenito C Poussin M Wang LP Ji C. *In vivo* persistence, tumor localization, and antitumor activity of car-engineered T cells is enhanced by costimulatory signaling through Cd137 (4-1bb). Cancer Res (2011) 71(13):4617–27. doi: 10.1158/0008-5472.Can-11-0422 PMC4140173 21546571

[60] Chmielewski M Hombach AA Abken H. Cd28 cosignalling does not affect the activation threshold in a chimeric antigen receptor-redirected T-cell attack. Gene Ther (2011) 18(1):62–72. doi: 10.1038/gt.2010.127 20944680

[61] Xie ZR Chen J Wu Y. Linking 3d and 2d binding kinetics of membrane proteins by multiscale simulations. Protein Sci (2014) 23(12):1789–99. doi: 10.1002/pro.2574 PMC4253819 25271078

[62] Boise LH Minn AJ Noel PJ June CH Accavitti MA Lindsten T. Cd28 costimulation can promote T cell survival by enhancing the expression of bcl-xl. Immunity (1995) 3(1):87–98. doi: 10.1016/1074-7613(95)90161-2 7621080

[63] Fang W Nath KA Mackey MF Noelle RJ Mueller DL Behrens TW. Cd40 inhibits B cell apoptosis by upregulating Bcl-Xl expression and blocking oxidant accumulation. Am J Physiol (1997) 272(3 Pt 1):C950–6. doi: 10.1152/ajpcell.1997.272.3.C950 9124531

[64] Haselager M Thijssen R West C Young L Van Kampen R Willmore E. Regulation of bcl-xl by non-canonical nf-Kb in the context of cd40-induced drug resistance in cll. Cell Death Differ (2021) 28(5):1658–68. doi: 10.1038/s41418-020-00692-w PMC8167103 33495554

[65] Viola A Lanzavecchia A. T cell activation determined by T cell receptor number and tunable thresholds. Science (1996) 273(5271):104–6. doi: 10.1126/science.273.5271.104 8658175

[66] Abu-Shah E Trendel N Kruger P Nguyen J Pettmann J Kutuzov M. Human Cd8(+) T cells exhibit a shared antigen threshold for different effector responses. J Immunol (2020) 205(6):1503–12. doi: 10.4049/jimmunol.2000525 PMC7477745 32817332

[67] Nguyen J Pettmann J Kruger P Dushek O. Quantitative contributions of Tnf receptor superfamily members to Cd8(+) T-cell responses. Mol Syst Biol (2021) 17(11):e10560. doi: 10.15252/msb.202110560 34806839 PMC8607805

