# Supplementary figures and images for: Signaling via a CD28/CD40 chimeric costimulatory antigen receptor (CoStAR™), targeting folate receptor alpha, enhances T cell activity and augments tumor reactivity of tumor infiltrating lymphocytes
Source: Front Immunol. 2023 Nov 7;14:1256491. doi: 10.3389/fimmu.2023.1256491 (PMC10664248; doi:10.3389/fimmu.2023.1256491)

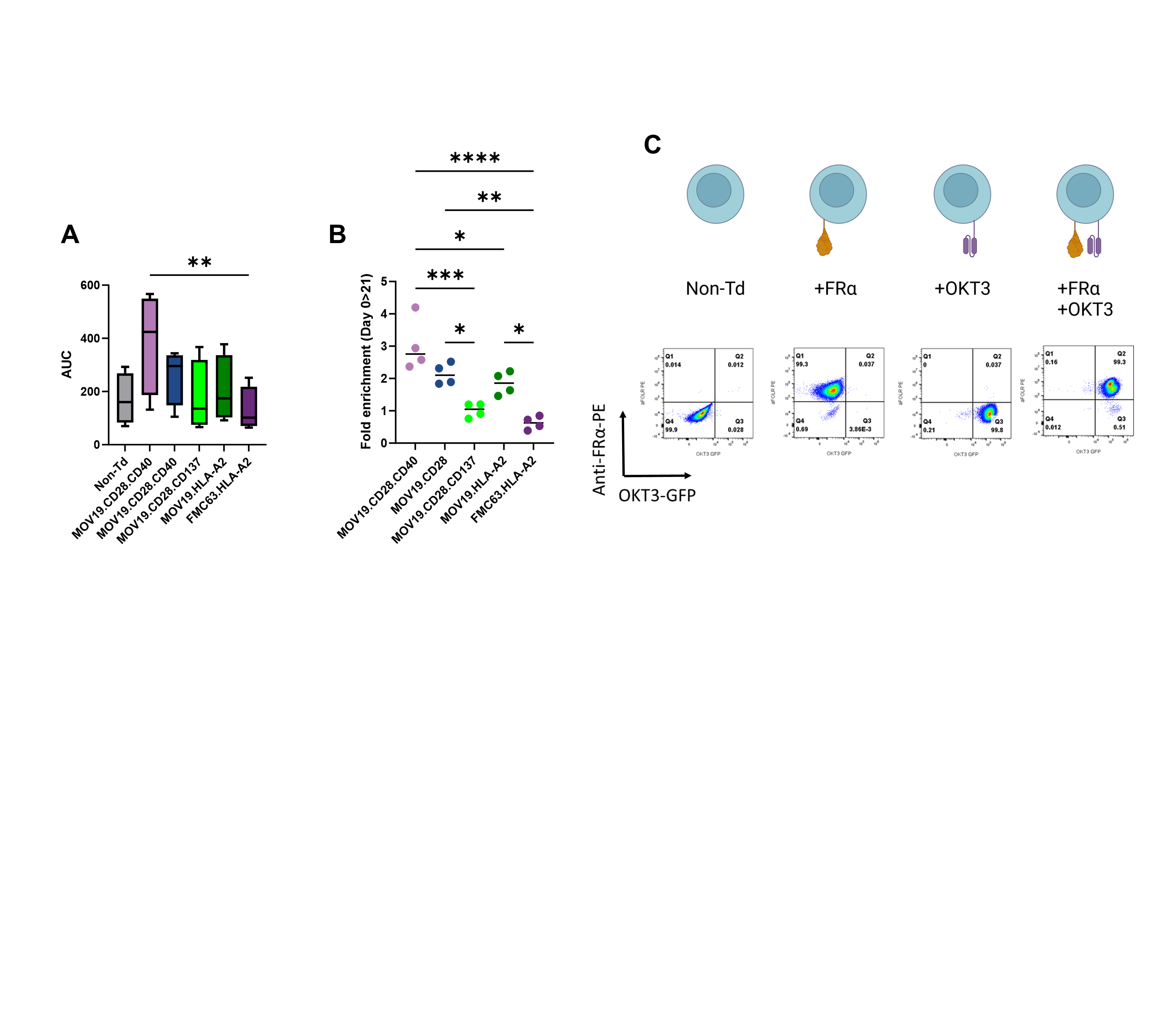

Supplement: Supplementary Figure 1 — A CD28.CD40-based CoStAR offers optimal antigen mediated costimulation of T cells. (A) T cells from four healthy donors were transduced with the indicated CoStARs and incubated with OvCAR-3.OKT3 at an E:T of 8:1 and proliferation of T cells (A) and CoStAR+ enrichment (B) monitored over four successive restimulations with additional tumor cells. Area under the curve (AUC) for expansion was calculated for each construct and significance calculated using a Friedman test: ** P <0.01. Fold enrichment of cells expressing each CoStAR was determined between days 0 and 21 and significance calculated using a one-way ANOVA with Tukey’s multiple comparison test: * P <0.05, ** P <0.01, *** P <0.001, **** P<0.0001. (C) BA/F3 cells were left non-transduced or engineered to express a membrane anchored OKT3 scFv and/or human FRα to provide signals 1 and/or 2 respectively. Flow cytometric analysis performed by staining with anti-FRα-PE or detection of OKT3 fluorescent tag (GFP). Figure created with Biorender.com. [file Image_1.tif]

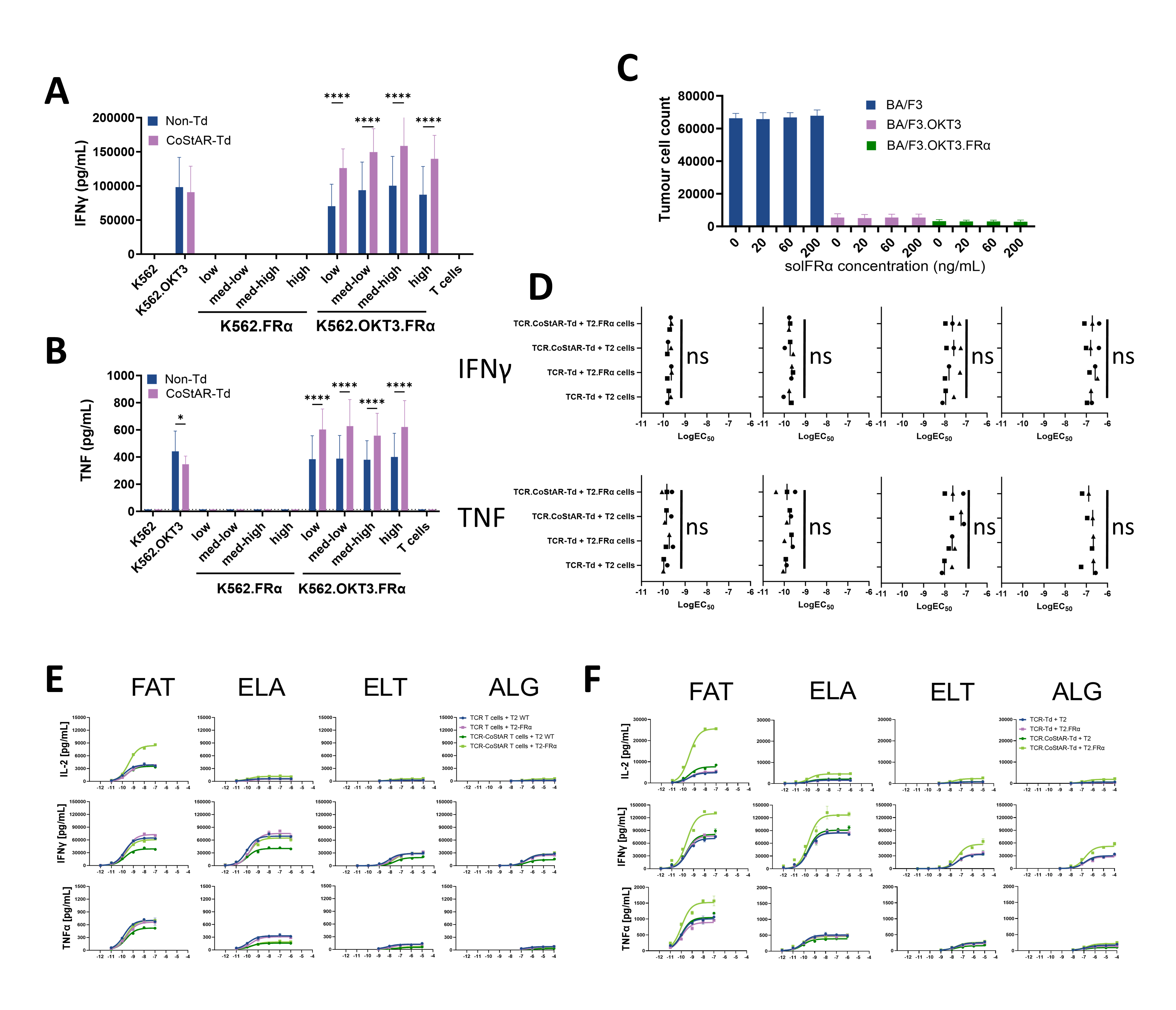

Supplement: Supplementary Figure 2 — CoStAR engineered cells respond to membrane bound, but not soluble, FRα; and in a dose dependent manner upon signal 1 engagement. Non-transduced (Non-Td) and CoStAR transduced (CoStAR-Td) T cells were cocultured at different effector: target (E:T) ratios with K562 parental, K562.OKT3, K562.FRα (low, med-low, med-high, or high), K562.OKT3.FRα (low, med-low, med-high, or high) cells. IFNγ (A) and TNF (B) secretion was measured after a 24-hour coculture. Averages from 3 donors in duplicate shown. * P <0.05; **** P <0.0001 by two-way ANOVA with Sidaks multiple comparison. (C) CoStAR-Td T cells were cocultured with BA/F3, BA/F3.OKT3 or BA/F3.OKT3.FRα cells at a 1:1 E:T in the presence of varying concentrations of soluble FRα (solFRα) protein ratio and residual tumor cells counted after 20 hours via murine anti-CD45 staining. (D) LogEC50 best-fit values were generated by fitting a dose-response curve to cytokine secretion data after T cell coculture with peptide-loaded T2 cell lines. Differences in LogEC50 values between TCR-Td T cells cocultured with T2 parental or T2.FRα both loaded with peptides (signal 1 only) and TCR.CoStAR-Td T cells cocultured with peptide-loaded wild type T2 (signal 1 only) compared with TCR.CoStAR-Td T cells cocultured with peptide-loaded T2.FRα (signals 1 and 2) were evaluated. LogEC50 best-fit values were calculated from secretion of IFNγ and TNF Averages from 2 technical replicates in 3 donors were analyzed by Friedman statistical test with Dunn’s multiple comparisons in Graphpad Prism 9.3.0. TCR-Td T cells (signal 1 only) or TCR.CoStAR-Td T cells (signals 1 and 2) were cocultured with parental T2 or T2.FRα, supernatants were collected after 20 hours of coculture and IL-2, IFNγ and TNF secretion evaluated by MSD assay. Two representative donors of three shown (E, F) performed in duplicate. [file Image_2.tif]

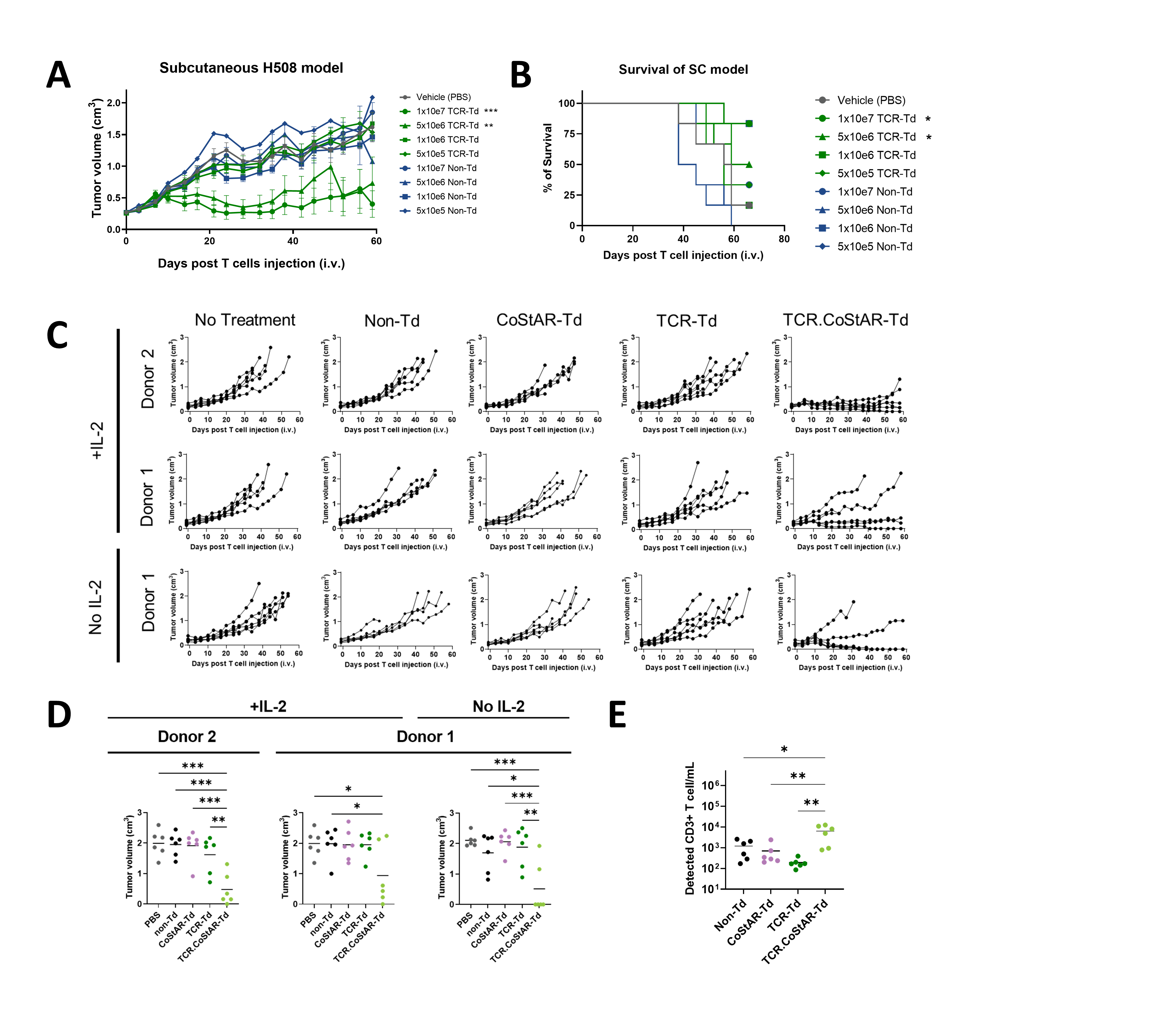

Supplement: Supplementary Figure 3 — CoStAR enhances T cell persistence and tumor control in a murine xenograft model even in the absence of exogenous IL-2 infusions. Mice were subcutaneously implanted with H508 tumors and administered with varying numbers of non-transduced or CEA-TCR engineered T cells at the indicated numbers. (A) Tumor volumes were measured by digital caliper measurements up to day 58. (B) Survival of animals was also monitored. (C) Tumor volumes were measured twice weekly by digital caliper measurements up to day 58. (D) Comparison of tumor volumes at day 58 in treatment groups from 2 donors with, and one donor without, administration of supportive IL-2. (E) Flow cytometric assessment of CD3+ T cells/mL in mouse tail-vein bleeds in all T cell groups with or without supportive IL-2 on day 14. Mean and individual data points shown. CD3+ levels were compared using one-way ANOVA with Tukey’s multiple comparison. To compare tumor volume mixed-effects model with Tukey’s multiple comparisons test was performed* P <0.05 (No treatment & Non-Td Vs TCR.CoStAR-Td) ** P <0.01 (TCR-Td & CoStAR-Td vs TCR.CoStAR-Td). To compare survival a log-rank test with Bonferroni correction for multiple corrections was used to determine the adjusted significance threshold, * P <0.05 (Non-Td & CoStAR Td vs TCR.CoStAR-Td), ** P <0.01. [file Image_3.tif]

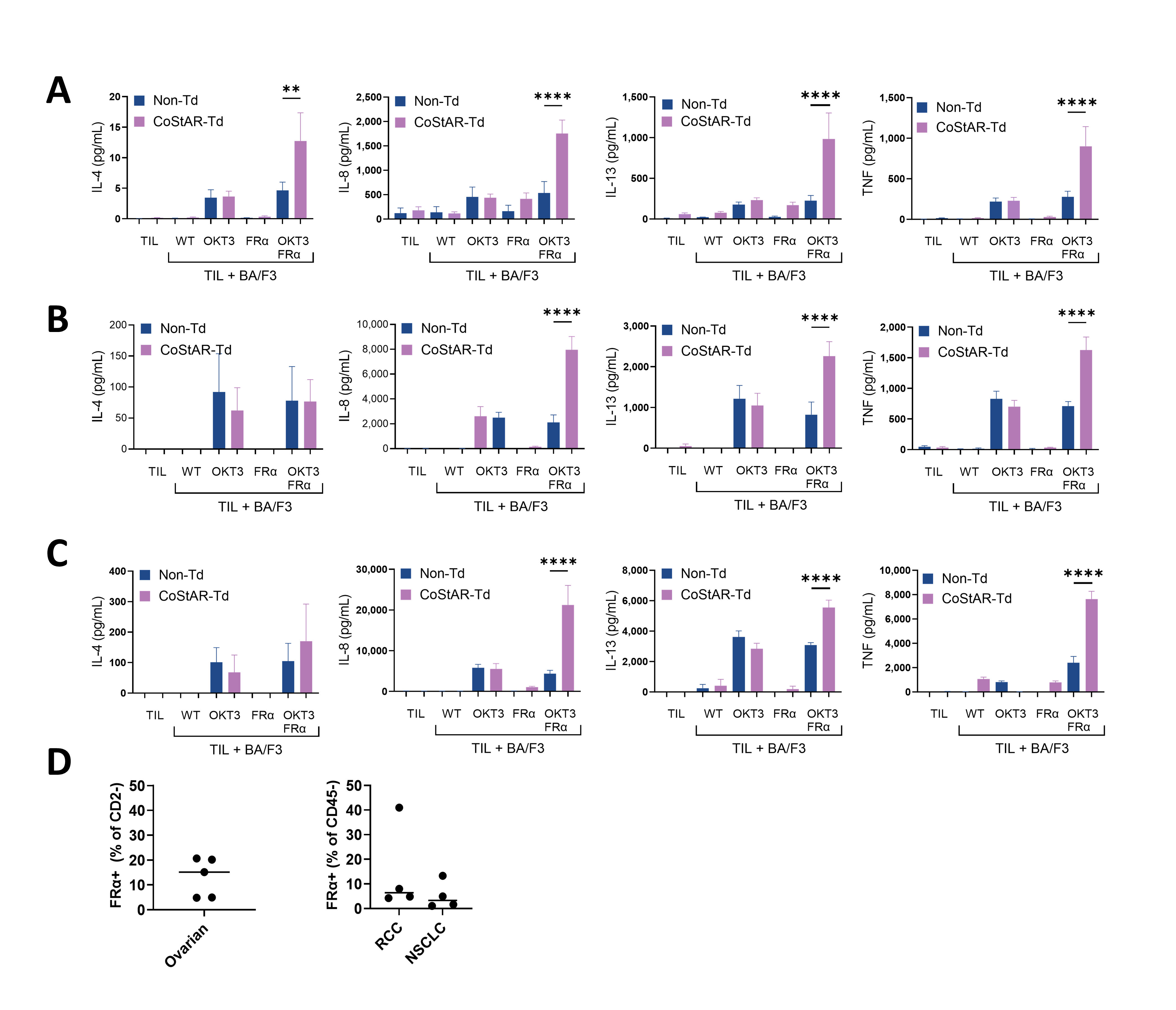

Supplement: Supplementary Figure 4 — CoStAR can be efficiently expressed in TIL and enhances effector function in response to autologous tumor. TIL from ovarian (A), renal (B) and non-small cell lung (C) were successfully transduced with CoStAR and cocultured with WT BA/F3 or BA/F3 cells expressing OKT3, FRα or OKT3 + FRα before measurement of IL-4, IL-8, IL-13 and TNF (A–C). FRα expression was assessed by flow cytometry in the CD2- population of ovarian digested tumor or CD45- population of renal and non-small cell lung cancer digests (D). * P <0.05, ** P <0.01 by one-way ANOVA with Sidak’s multiple comparisons test. [file Image_4.tif]
