# Supplementary material for: Signaling via a CD28/CD40 chimeric costimulatory antigen receptor (CoStAR™), targeting folate receptor alpha, enhances T cell activity and augments tumor reactivity of tumor infiltrating lymphocytes
Source: Front Immunol. 2023 Nov 7;14:1256491. doi: 10.3389/fimmu.2023.1256491 (PMC10664248; doi:10.3389/fimmu.2023.1256491)
Supplement: Supplementary file 5 [file Table_1.docx]

| Antigen/reagent | Fluor. | Supplier | Code |
| --- | --- | --- | --- |
| Anti-human | | | |
| CD2 | PE | Biolegend | 300208 |
|  | PE/Cy7 | Biolegend | 300222 |
|  | BB700 | BD | 566519 |
| CD3 | AF700 | Biolegend | 300424 |
|  | BV711 | Biolegend | 344838 |
|  | APC | Biolegend | 300439 |
|  | PerCP-Cy5.5 | Biolegend | 300430 |
| CD4 | FITC | Biolegend | 317408 |
|  | BUV737 | BD | 612748 |
|  | BUV805 | BD | 612887 |
|  | PE/Cy7 | Biolegend | 357410 |
| CD8 | PE/Cy7 | Biolgened | 344712 |
|  | BUV395 | BD | 563795 |
|  | APC-Vio770 | Miltenyi | 130-113-155 |
| CD45 | BV785 | Biolegend | 304048 |
| CD45RA | APC | Biolegend | 304112 |
| CD45RA | BV510 | Biolegend | 304142 |
| CD45RO | BV785 | Biolegend | 304234 |
| CD56 | BUV395 | BD | 563554 |
| CD95 | BV650 | Biolegend | 305642 |
| CCR7 | BV421 | Biolegend | 353208 |
| FRα | PE | Biolegend | 908304 |
| TCRαβ | APC/Cy7 | Biolegend | 306728 |
| TCRαβ | BV421 | Biolegend | 306722 |
| TCRγδ | BUV395 | Biolegend | 564155 |
| IgG | PE | Sigma-Aldrich | P9170-0.5ml |
| PD-1 | APC | Biolegend | 329908 |
| Anti-mouse | | | |
| CD45 | BV785 | Biolegend | 103149 |
| TCRβ | BV421 | Biolegend | 109230 |
| TCRβ | PE-Cy7 | Biolegend | 109222 |
| IgG1 | PE | Biolegend | 406607 |
